# Flow Chemistry for Synthesis of 2-(C-Glycosyl)acetates from Pyranoses via Tandem Wittig and Michael Reactions

**DOI:** 10.1021/acs.oprd.3c00414

**Published:** 2024-02-28

**Authors:** Jack J. Bennett, Paul V. Murphy

**Affiliations:** † School of Biological and Chemical Sciences, University of Galway, University Road, Galway H91 TK33, Ireland; ‡ SSPC − SFI Research Centre for Pharmaceuticals, University of Galway, University Road, Galway H91 TK33, Ireland

**Keywords:** continuous flow processing, tandem reaction, Wittig reaction, Michael reaction, 1,4-conjugate addition, C-glycosides

## Abstract

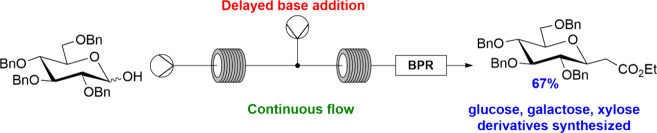

C-Glycosyl compounds (C-glycosides) are a class of saccharide derivatives with improved stability over their O-linked counterparts. This paper reports the synthesis of several *trans*-2-(C-glycosyl)acetates via a tandem Wittig–Michael reaction from pyranoses (cyclic hemiacetals) using continuous flow processing, which gave improvements compared to reactions conducted in round-bottom flasks. Products were isolated in yields of >60% from reactions of benzyl-protected xylopyranoses, glucopyranoses, and galactopyranoses at higher temperatures and pressures, which were superior to yields from batch procedures. A two-step procedure involving the Wittig reaction followed by Michael reaction (intramolecular oxa-Michael) of the unsaturated ester obtained in the presence of DBU was developed. Reactions of protected mannopyranose gave low yields in corresponding reactions in flow due to competing C-2 epimerization.

## Introduction

Glycosides are ubiquitous molecules that play essential roles in numerous biological pathways. C-Glycosyl compounds (C-glycosides) are medicinally important glycomimetics, in part due to their resistance toward hydrolysis compared to the corresponding *O*-glycosides, which do not exhibit the same stability.^1,2^ C-Glycosides have been explored widely in drug discovery^3−7^ and in materials chemistry,^8,9^ and therefore, development of synthetic routes toward these structures is important.

Several routes to C-glycosides have been reported in the literature and comprehensively reviewed by Yang and Yu.^2^ Methods for synthesis of C-glycosides include substitution reactions via glycosyl cationic/anionic/radical species,^10−13^ coupling reactions promoted by transition metal complexes,^14−16^ rearrangements,^17,18^ and further synthetic elaboration after formation of the C–C bond to the anomeric carbon.^19,20^

**Scheme 1:**
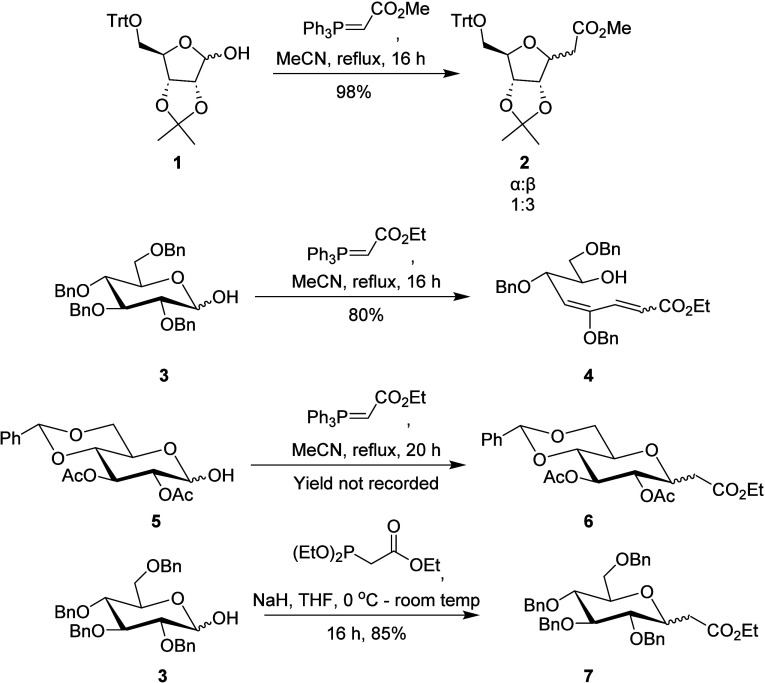
Previously Reported Batch Methods for the Synthesis of C-Glycosyl Esters^19,22,23^

Pyranoses, which are hemiacetals with a latent aldehyde, can undergo the Wittig or Horner–Wadsworth–Emmons (HWE) reaction, and when these reactions can be used in conjunction with conjugate addition (Michael reaction), 2-(glycosyl)acetates can be formed (Scheme 1).^19,21^ The tandem Wittig–Michael procedure, the subject of this paper, was studied for synthesis of C-nucleosides **2** via 2,3-isopropylidene sugars **1** by Moffatt and co-workers,^22^ who obtained anomeric mixtures (Scheme 1). Subsequently, Nicotra et al.^23^ studied the Moffatt procedure on pyranoses and found the formation of a high yield of an elimination product **4** from glucopyranose **3**, although they did report a successful C-glycoside synthesis of **6** by reaction of 4,6-*O*-benzylidene-2,3-di-*O*-acetyl-d-glucopyranose **5** (Scheme 1), although a yield was not given.^23^ The HWE-based procedure was originally used by Monti et al. to synthesize C-mannopyranosides and glucopyranosides like **7**, where they noted that use of the Moffatt procedure was unsuccessful.^24^ Later, Arya et al. prepared similar C-glycosides to Monti et al. en route to macrocycles via the HWE procedure.^19,21−24^

Continuous flow chemistry is an evolving field of organic synthesis in which reactions are carried out in continuous streams through narrow tubing/channels in modular systems. In comparison with batch, flow systems have significantly higher surface area to volume values for standard mesofluidic reactors, with further improvements possible with microfluidic systems. In addition, flow systems have inherently higher pressures over batch, controlled through use of a back-pressure regulator (BPR). These factors of flow chemistry provide numerous benefits over batch techniques, such as improved safety, greater heat transfer, improved mixing profiles, the potential for automation, and improved scalability.^25−27^ The advent of commercial laboratory-scale flow reactors has also made flow chemistry more accessible to synthetic chemists and boosted growth of this field over the past two decades.^28−33^

Continuous flow chemistry has been used in several areas of glycochemistry, such as in the study of glycosylation reactions and the synthesis of glycans.^34−37^ In addition, it has found uses in protecting group chemistry applied in glycomolecule synthesis, including global deprotections.^38−40^ Flow chemistry has also been applied in C-glycoside synthesis, for example, in the synthesis of dapagliflozin^41^ (a drug for Type 2 diabetes) and remdesivir^42^ (an antiviral used to treat COVID-19). However, use of continuous flow chemistry has been mainly limited to synthesis of C-aryl glycosides,^43,44^ highlighting the need to investigate its suitability to give C-alkyl and related glycosides. We recently reported its use in improvement of the synthesis of iminosugars.^45^ Herein we show benefits of using flow chemistry to obtain 2-(C-glycosyl)acetates via the tandem Wittig–Michael approach.

## Results and Discussion

### 2.1 Reactions of 2,3,4-Tri-*O*-benzyl-d-xylopyranose in Absence of Base

**Scheme 2:**
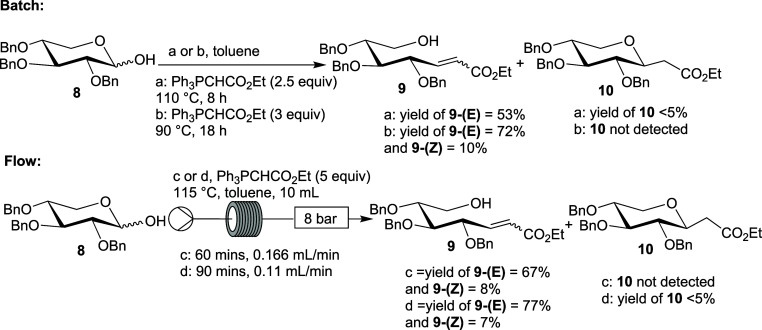
Wittig Reaction of Xylopyranose **8** in Batch and Flow

The preparation of 2,3,4-tri-*O*-benzyl-d-xylopyranose (**8**) was carried out as described previously.^46^ Initially, attempts to improve the Wittig olefination of **8** with (carbethoxymethylene)triphenylphosphorane were carried out both in batch and under continuous flow conditions (Scheme 2). Only products **9** (*E* and *Z* isomers) were obtained in 82% yield after reaction with 3 equiv of Wittig reagent in toluene in batch; **9** could be obtained in reduced reaction time by continuous flow in similar yield and stereoselectivity.

With reliable procedures to generate the Wittig product mixture, **9-(E)** and **9-(Z)**, our attention turned toward varying the reaction conditions to promote conjugate addition from **9** to form β-C-glycosylacetate **10** (Table 1). Flow techniques allow safe investigation of temperatures exceeding the atmospheric boiling point of a solvent, which is possible due to the use of reactors with high resistance to pressure. Thus, using the Vapourtec R-Series system, xylopyranose **8** and Ph_3_PCHCO_2_Et were premixed in toluene and injected into the system via sample loop, and the temperature was increased from 115 to 125 °C; use of a residence time of 45 min in a 5 mL coiled-tube reactor showed a small improvement in the formation of Michael product **10**, from a batch yield of <5% to 17% from flow. A subsequent flow reaction at 150 °C with 40 min residence time gave **10** in 32% yield. See Table S1 for ratios of crude products from flow and batch reactions from xylose and glucose derivatives.

**Table 1:**
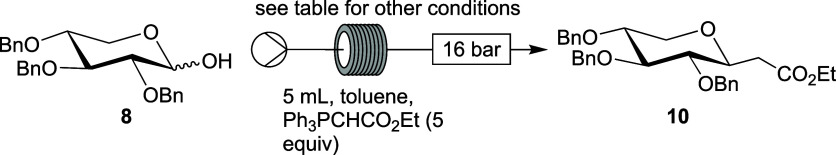
Formation of **10** under Continuous Flow Conditions in Absence of Base

entry	temperature (°C)	time (min)	flow rate (mL/min)	isolated yield of **10** (%)
1	125	45	0.11	17
2	150	40	0.125	32

### 2.2 Reactions of 2,3,4,6-Tetra-*O*-benzyl-d-glucopyranose in Absence of Base

2,3,4,6-Tetra-*O*-benzyl-d-glucopyranose (**3**) was subjected to flow reactions using temperatures similar to those used for **8**, but these resulted in very low yields of **7b** (the β anomer of **7**), so the temperature was increased to over 200 °C, giving the desired product **7b** in low yield (Table 2). The procedure involved premixing glucopyranose **3** with Ph_3_PCHCO_2_Et in toluene, injecting the mixture into the flow system, and then passing the mixture through the 5 mL coiled-tube reactor. Initially, 3 equiv of the Wittig reagent was used with a residence time of 50 min at 220 °C, which gave **7b** in 35% yield. Increasing the residence time and temperature to 60 min and 230 °C, respectively, gave a similar yield of 30%. Use of a 60 min residence time at 210 °C with 5 equiv of Ph_3_PCHCO_2_Et afforded **7b** in 42% yield.

**Table 2:**
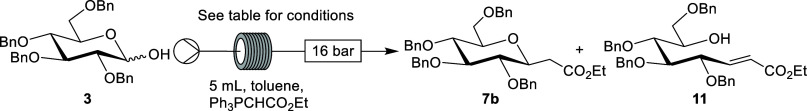
Isolation of Michael Addition Product **7b** and Wittig Intermediate **11** in Absence of Base

					isolated yields (%)	
entry	equiv of Ph_3_PCHCO_2_Et	temperature (°C)	time (min)	flow rate (mL/min)	**7b**	**11**
1	3	220	50	0.1	35	14
2	3	230	60	0.083	30	12
3	5	210	60	0.083	42	10

### 2.3 Reactions of 2,3,4,6-Tetra-*O*-benzyl-d-glucopyranose and 2,3,4-Tri-*O*-benzyl-d-xylopyranose in the Presence of DBU

It was envisaged that addition of a base after initial alkene formation would improve the Michael reaction. Thus, an alternative continuous flow setup was used, as summarized in Table 3. The pyranose was premixed with Ph_3_PCHCO_2_Et and then passed through a high-temperature 5 mL reactor, after which DBU was injected and mixed with the reaction mixture before it passed through a 10 mL coiled-tube reactor. Using the conditions shown in Table 3, entry 1, the xylopyranose reactant **8** gave **10** in 45% yield, which was a similar yield to the best attempt in the absence of base; the α-Michael product **12** was isolated as a minor product. A potential reason for a similar yield is base-promoted retro-Michael additions as reported previously, which might be more competitive at higher temperatures.^47^ Hence, the use of lower temperatures in reactors 1 and 2 (180 and 130 °C, respectively) and lengthened residence time (45 min per reactor) led to improvement in the yields of β and α Michael products **10** and **12** to 55% and 15%, respectively (entry 2). The yield of 2-(β-C-glucopyranosyl)acetate **7b** was also improved with this approach. In the first attempt, glucopyranose **3** and Ph_3_PCHCO_2_Et were passed through a 5 mL coiled-tube reactor for 65 min at 200 °C, followed by delayed addition of DBU and further passage through a 10 mL coiled-tube reactor for 65 min at 145 °C, giving the product **7b** in 63% yield. A subsequent flow reaction using a residence time of 40 min in each reactor and a higher temperature of 210 °C in the first reactor gave **7b** in similar 67% isolated yield. While the yield is lower than for the tandem batch-based HWE–Michael reaction carried out by Arya et al.,^19^ the flow reaction takes less time, and the stereoselectivity is higher. The Wittig–Michael approach is shown to be feasible in continuous flow, in contrast with that reported in batch by Nicotra et al. Reactions with unprotected d-glucose were also investigated but failed to produce any useful product as indicated by analysis of the crude product by ^1^H/^13^C NMR spectroscopy, confirming the requirement for protecting groups in this case.

**Table 3:**
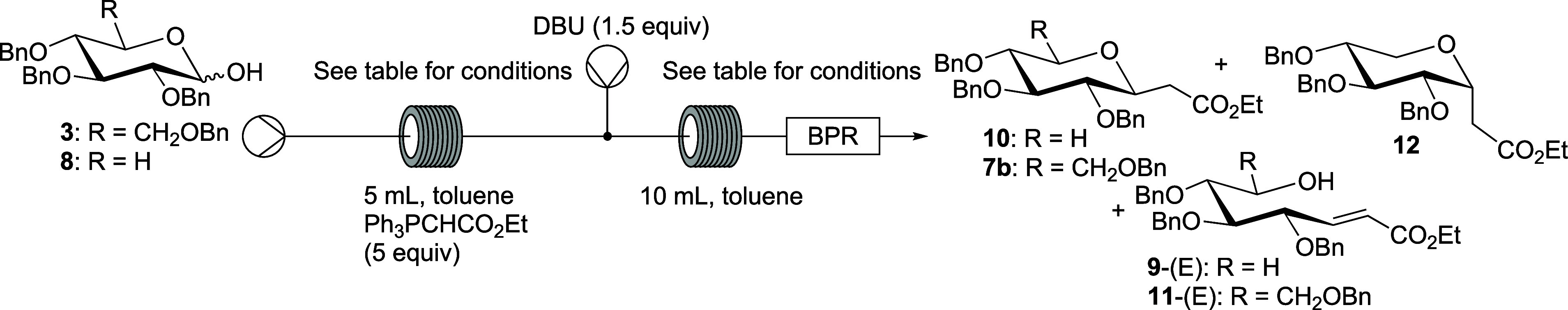
DBU-Improved Formation of C-Glycosyl Compounds from **3** and **8**

		reactor 1		reactor 2				products (yields, %)		
entry	reactant	temperature (°C)	time (min)	temperature (°C)	time (min)	flow rate per pump (mL/min)	BP (bar)	β	α	Wittig product
1	**8**	210	40	145	40	0.125	16	**10** (45)	**12** (8)	**9-(E)** (16)
2	**8**	180	45	130	45	0.11	8	**10** (55)	**12** (15)	**9-(E)** (10)
3	**3**	200	65	145	65	0.077	8	**7b** (63)	–	**11-(E)** (9)
4	**3**	210	40	145	40	0.125	16	**7b** (67)	–	**11-(E)** (7)

### 2.4 Reactions of 2,3,4,6-Tetra-*O*-benzyl-d-galactopyranose and 2,3,4,6-Tetra-*O*-benzyl-d-mannopyranose in the Presence of DBU

Next, we investigated the tandem Wittig–Michael reaction of other pyranoses (Table 4). Employing the two-step approach with delayed addition of DBU, the C-galactopyranosyl compound **14** was formed from **13**(48) in 43% yield as a mixture of anomers using temperatures of 200 and 145 °C in reactors 1 and 2, respectively (Table 4, entry 1). Lowering the temperatures of reactor 1 and 2 to 180 and 130 °C, respectively, and decreasing the residence time per reactor to 45 min gave **14** as a mixture of anomers in an improved yield of 60%.

**Table 4:**
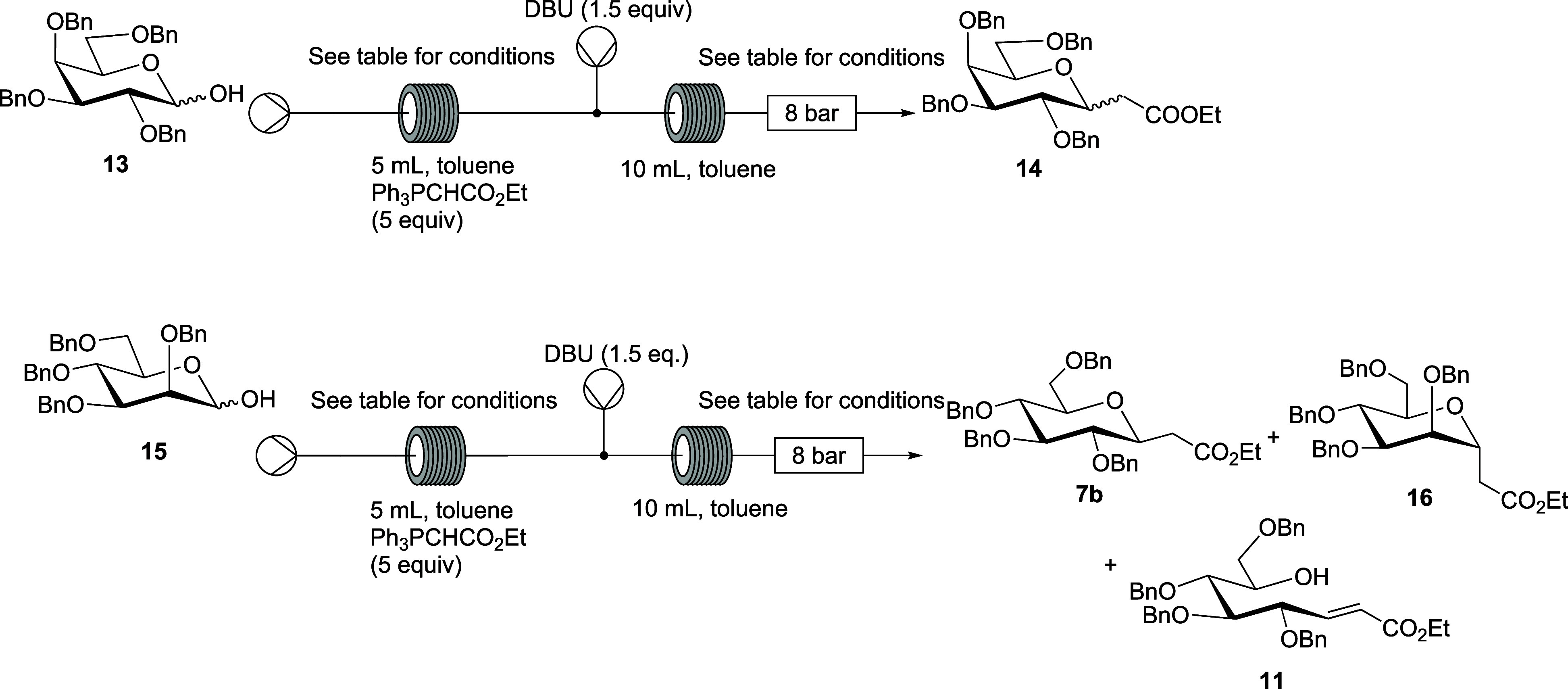
Reactions of **13** and **15**

		Reactor 1		Reactor 2				products (isolated yields, %)		
entry	reactant	temperature (°C)	time (min)	temperature (°C)	time (min)	flow rate per pump (mL/min)	BP (bar)	C-glycosyl product		Wittig product
1	**13**	200	65	145	65	0.077	8	**14**, α:β 0.5:1 (43)		–
2	**13**	180	45	130	45	0.11	8	**14**, α:β 0.5:1 (60)		–
3	**15**	200	65	130	65	0.077	8	**7b** (11)	**16** (31)	**11-(E)** (45)
4	**15**	180	45	130	45	0.11	8	**7b** (0)	**16** (26)	**11-(E)** (35)

Similar flow conditions were used for the mannopyranose reactant **15**,^49^ with a lower heat of 130 °C in reactor 2 giving the α-C-mannopyranoside **16** in 31% yield after column chromatography, with traces of β product also present. The lower yields are a result of C-2 epimerization of the mannopyranose reactant **15**,^21^ with the corresponding Michael addition product **7b** and intermediate Wittig product **11** formed in 11% and 45% yield, respectively. A subsequent reaction using a lower temperature of 180 °C in reactor 1 and a reduced residence time of 45 min per reactor yielded the α-C-mannopyranosyl compound **16** in 26% yield and the gluco-configured Wittig product **11** in 35% yield.

### 2.5 Reduction of Products to Facilitate Compound Characterization

**Scheme 3:**
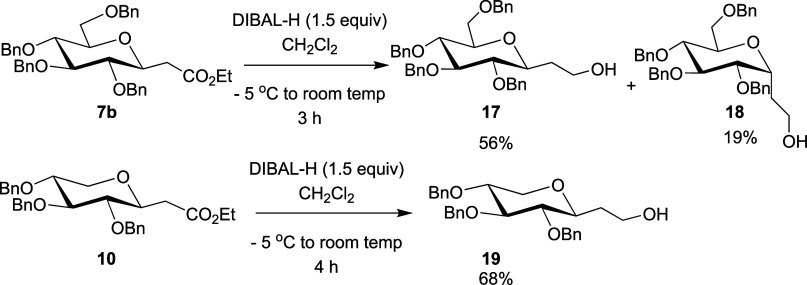
Reduction of Esters **7b** and **10** in Batch Using DIBAL-H

To assist with analysis of the stereochemical configuration of the C-glycosyl acetates formed and due to relative instability of the products, the reduction of **7b** and **10** to the respective more stable primary alcohols was carried out (Scheme 3). Reaction of **7b** with DIBAL-H at −5 °C to room temperature under batch conditions gave β alcohol **17** in 56% yield, with the minor α product **18** also formed in 19% yield. This epimerization is likely due to the reversibility of the Michael addition reaction under basic conditions. Previous reported attempts of this reaction at −78 °C gave the β product exclusively.^50^ Using the same conditions with a longer reaction time of 4 h, 2-(β-C-xylopyranosyl)acetate **10** was converted to alcohol **19** in 68% yield. NMR analysis of **17**–**19** (see the Experimental Section) contributed to confirming the stereochemical assignment for **10**, by comparing its data with those of **7b** reported previously.

## 3 Conclusions

The tandem Wittig–Michael addition reaction for formation of 2-(C-glycosyl)acetates was carried out from protected pyranoses using continuous flow techniques. Reproducing access to the unsaturated esters from the Wittig reaction was found to be straightforward under conditions of continuous flow. A protocol is given involving addition of DBU which leads to improved synthesis of the C-glycosyl acetates from the unsaturated esters formed from the initial Wittig reaction. One reviewer commented that the production of triphenylphosphine oxide (TPPO) is an issue with Wittig reactions given that it can be difficult to separate from the alkene product; they indicated that the HWE alternative is superior given that it generates a water-soluble byproduct. We agree with the reviewer’s assessment and note that Antonio Rodríguez Hergueta recently addressed the removal of TPPO by precipitation with CaBr_2_, which was reported to be successful from toluene, used as a solvent in this research.^51^ The improvement to this reaction with continuous flow chemistry is clearly demonstrated herein, as in batch the tandem Wittig–Michael addition reaction leads to formation of side products or very low yields of C-glycosyl products.^23,24^

## 4 Experimental Section

### General

All analytical data for previously reported compounds were found to be in accordance with data reported in the literature, and citations are provided. All reagents used were obtained from commercial sources and used without further purification. TLC experiments were used to monitor reactions and were performed using aluminum sheets precoated with silica gel 60 (HF_254_, E. Merck, Merck KGaA, Darmstadt, Germany), with spots visualized by UV and charring with aqueous KMnO_4_ and vanillin. NMR spectra were processed and analyzed using MestReNova software (v14.0.0-23239, mestrelab.com, Barcelona, Spain). Chemical shifts are reported relative to internal Me_4_Si in CDCl_3_ (δ 0.0). CDCl_3_ (δ 77.16) signals were used for ^13^C experiments. Signals from ^1^H and ^13^C spectra were assigned using COSY, HSQC, and HMBC. *J* values are reported as observed. The IR spectra were obtained using a PerkinElmer Spectrum 100 FTIR spectrometer. High-resolution mass spectra were obtained using an Agilent UHPLC-MS. Chromatography was performed with silica gel 60 using cyclohexane and EtOAc. Reaction solvents were used as obtained from a Pure-Solv solvent purification system. Flow reactions were carried out using a Vapourtec R-Series reactor.

### 2,3,4-Tri-*O*-benzyl-d-xylopyranose (**8**)

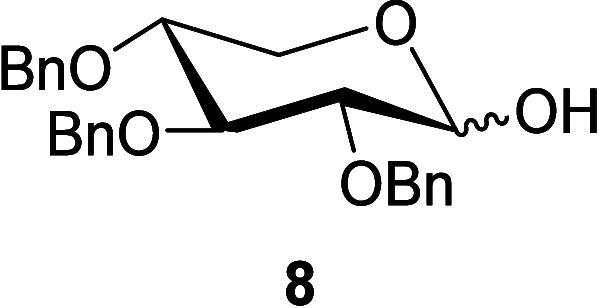



Compound **8** was prepared using previously reported methods.^46,52,53^d-Xylose (11.7 g, 0.078 mol) was dissolved in MeOH (150 mL), to which DOWEX 50WX8 (H^+^) (11.7 g) was added, and the mixture was stirred at reflux for 12 h. The mixture was filtered, and the solvents were removed under reduced pressure. The residue was redissolved in DMF (200 mL) and cooled to 0 °C. BnBr (32.4 mL, 0.27 mol) was added slowly, and the mixture was allowed warm to room temperature and stirred for 12 h. Water (50 mL) was added at 0 °C, and the aqueous layer was extracted with EtOAc (30 mL × 3). The combined organic fractions were washed with brine and dried over Na_2_SO_4_, and the solvent was removed under reduced pressure. Flash column chromatography (5% to 10% EtOAc in cyclohexane) yielded a mixture of methyl 2,3,4-tri-*O*-benzyl-α- and -β-d-xylopyranosides (27 g), which was redissolved in 1,4-dioxane (100 mL). AcOH (75 mL) and aqueous 2 M H_2_SO_4_ (25 mL) were charged to the flask, and the mixture was stirred at reflux for 16 h and then cooled to room temperature. H_2_O/hexane (8:1, 45 mL) was added, and the mixture was stirred for 30 min. The white precipitate obtained was filtered, washed with H_2_O (30 mL × 2), and crystallized from MeOH. The crystals, which were of the title compound, were separated, washed with H_2_O, and dried. The filtrate and mother liquor had their solvents removed under reduced pressure. Column chromatography (20% EtOAc in cyclohexane) of the residue gave a second batch of the title compound, which when added to the first batch of crystals gave **8** (14.7 g, 45% over three steps) as a mixture of anomers. All analytical data for **8** were in agreement with those previously reported.^52,54^

^1^H NMR (500 MHz, CDCl_3_) δ 7.37–7.27 (ms, 15H, aromatic), 5.11 (s, 1H, H-1α), 4.93–4.88 (ms, 4H, −OCH_2_Ph), 4.80–4.71 (ms, 4H, −OCH_2_Ph), 4.70–4.61 (ms, 3H, −OCH_2_Ph, H-1β), 3.95–3.90 (ms overlapped, 2H, H-5β, H-3α), 3.84 (t, *J* = 10.7 Hz, 1H, H-5α), 3.72–3.56 (ms overlapped, 4H, H-5′α, H-4α, H-3β, H-4β), 3.52 (dd, *J* = 9.0, 3.5 Hz, 1H, H-2α), 3.35–3.25 (ms overlapped, 2H, H-2β, H-5′β).

^13^C NMR (126 MHz, CDCl_3_) δ 138.6 (Ar Cα), 138.5 (Ar Cβ), 138.3 (Ar Cβ), 138.2 (Ar Cα), 138.1 (Ar Cβ), 137.8 (Ar Cα), 128.4–127.9 (Ar CH), 97.7 (C-1β), 91.5 (C-1α), 83.1 (C-3β), 82.3 (C-2β), 80.4 (C-3α), 79.4 (C-2α), 77.5 (C-4β), 77.4 (C-4α), 75.5 (−OCH_2_Phα), 74.8 (−OCH_2_Phβ), 73.4 (−OCH_2_Phα), 73.2 (−OCH_2_Phβ), 73.2 (−OCH_2_Phα), 63.7 (C-5β), 60.4 (C-5α).

### 2,3,4,6-Tetra-*O*-benzyl-d-mannopyranose (**15**)

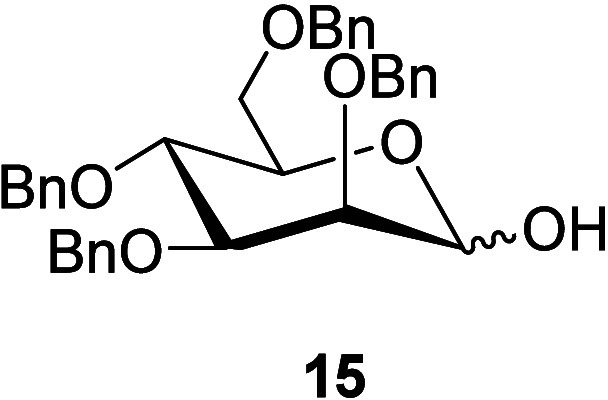



Compound **15** was prepared using previously reported methods.^55^ Methyl α-d-mannopyranoside (1.50 g, 0.0077 mol) was dissolved in DMF (30 mL) and cooled to 0 °C. NaH (1.11 g, 0.046 mol) was added, and the mixture was stirred for 30 min. BnBr (5.5 mL, 0.046 mol) was added dropwise at 0 °C, and the mixture was warmed to room temperature after 10 min. The mixture was stirred at room temperature for 8 h, after which it was cooled to 0 °C and quenched with addition of H_2_O (20 mL). The aqueous layer was extracted with EtOAc (25 mL × 2), and the combined organic fractions were washed with H_2_O (25 mL × 3) and brine (20 mL). The organic layer was dried over Na_2_SO_4_, and the solvents were removed under reduced pressure. The crude residue was redissolved in glacial AcOH (40 mL), to which aqueous 2 M H_2_SO_4_ (15 mL) was added. The mixture was refluxed at 90 °C for 16 h, after which it was cooled to room temperature. The mixture was diluted with water (40 mL) and extracted with EtOAc (25 mL × 3), and the combined organic fractions were washed with saturated aqueous NaHCO_3_ (30 mL × 2), H_2_O (30 mL), and brine (20 mL). The organic layer was dried over Na_2_SO_4,_ and the solvents were removed under reduced pressure. Column chromatography (25% EtOAc in cyclohexane) gave the hemiacetal **15** (0.81 g, 1.49 mmol, 19% over two steps) as a mixture of anomers (α:β 4:1). All analytical data were in agreement with those previously reported.^56^

^1^H NMR (400 MHz, CDCl_3_) δ 7.41–7.27 (ms, 20H), 7.20–7.15 (ms, 2H), 5.25 (d, *J* = 1.9 Hz, 1H, H-1α), 4.90 (d, *J* = 11.0 Hz, 1H, −CH_2_Bn), 4.73 (d, *J* = 2.8 Hz, 2H, −CH_2_Bn), 4.64 (apt s, 0.28 H, H-1β), 4.61 (apt s, 2H, −CH_2_Bn), 4.55 (d, *J* = 8.5 Hz, 2H, −CH_2_Bn), 4.51 (m, 1H, −CH_2_Bn), 4.06 (ddd, *J* = 9.8, 6.6, 2.1 Hz, 1H, H-5α), 3.97 (dd, *J* = 9.4, 3.1 Hz, 1H, H-3α), 3.86 (t, *J* = 9.7 Hz, 1H, H-4α), 3.79 (dd, *J* = 3.1, 1.9 Hz, 1H, H-2α), 3.71 (m, 1H, H-6aα), 3.68 (dd, *J* = 10.2, 6.5 Hz, 1H, H-6bα), 3.59 (dd, *J* = 9.4, 2.8 Hz, 0.28 H, H-3β), 3.46 (dt, *J* = 9.4, 3.8 Hz, 0.27 H, H-5β).

^13^C NMR (126 MHz, CDCl_3_) δ 138.6 (Ar C), 138.5 (Ar C), 138.2 (Ar C), 138.2 (Ar C), 138.1 (Ar C), 138.0 (Ar C), 128.4 (Ar CH), 128.2 (Ar CH), 128.1 (Ar CH), 128.0 (Ar CH), 127.9 (Ar CH), 127.7 (Ar CH), 127.6 (Ar CH), 127.6 (Ar CH), 93.9 (C-1β), 92.7 (C-1α), 83.1 (C-3β), 79.8 (C-3α), 76.0 (C-2β), 75.3 (C-4α), 75.2 (C-5β), 75.1 (−CH_2_Bn), 75.0 (C-2α), 74.7 (−CH_2_Bn), 73.6 (−CH_2_Bn), 73.3 (−CH_2_Bn), 72.7 (−CH_2_Bn), 72.2 (−CH_2_Bn), 71.4 (C-5α), 69.7 (C-6α), 69.2 (C-6β).

### 2,3,4,6-Tetra-*O*-benzyl-d-galactopyranose (**13**)

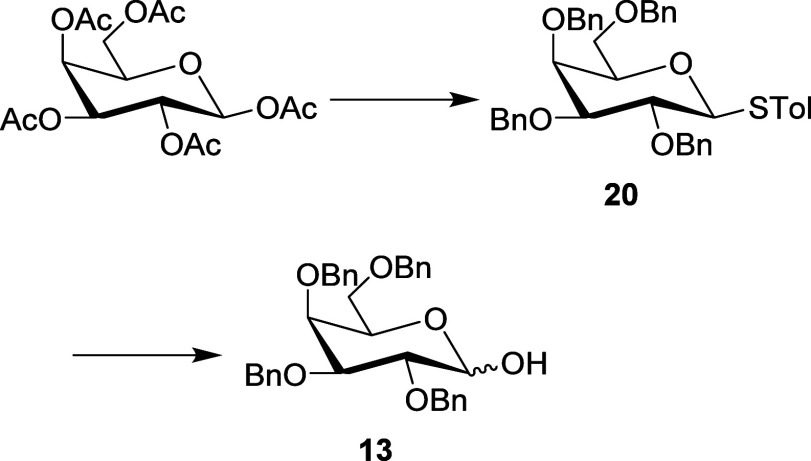



Compound **13** was prepared using previously reported methods.^48,57^ Penta-*O*-acetyl-β-d-galactopyranose (0.5 g, 1.28 mmol) was dissolved in dry DCM (20 mL) to which 4-methylbenzenethiol (0.23 mL, 1.92 mmol) was charged. The mixture was cooled to 0 °C, and BF_3_OEt_2_ (0.24 mL, 1.92 mmol) was added dropwise. The mixture was warmed to room temperature and stirred for 2 h. The mixture was quenched with cold H_2_O (10 mL), and the layers were separated. The aqueous layer was extracted with DCM (10 mL), and the combined organic layers were washed with cold saturated aqueous NaHCO_3_ (20 mL) and brine (10 mL). The organic layer was dried over Na_2_SO_4_, and the solvents were removed under reduced pressure. The residue was redissolved in dry MeOH (15 mL), and 1 M NaOMe (0.26 mL, 0.26 mmol) was charged at room temperature. The mixture was stirred for 1 h, quenched with Amberlite IRC-120 (H+), and filtered. The solvents were removed under reduced pressure, and the residue was redissolved in dry DMF (20 mL) and cooled to 0 °C. NaH (0.30 g, 7.68 mmol) was added, and the mixture was stirred for 30 min and then cooled to 0 °C. BnBr (0.91 mL, 7.68 mmol) was added dropwise, and the mixture was warmed to room temperature after 10 min. The mixture was stirred for 16 h and quenched with cold H_2_O (20 mL). The mixture was diluted with EtOAc, and the layers were separated. The organic layer was washed with an excess of H_2_O (20 mL × 3) and brine (10 mL). The organic layer was dried over Na_2_SO_4_, and the solvents were removed under reduced pressure. Column chromatography (10% EtOAc in cyclohexane) gave the intermediate **20** (0.34 g, 0.52 mmol, 41% over three steps) as a white solid. ^1^H NMR data for **20** were in agreement with those previously reported.^57^

^1^H NMR (400 MHz,CDCl_3_) δ 7.45 (d, *J* = 8.2 Hz, 2H, −SPh), 7.40–7.25 (ms, 20H, Ar H), 6.97 (d, *J* = 8.0 Hz, 2H, −SPh), 4.95 (dd, *J* = 9.9, 4.0 Hz, 1H, H-1), 4.77 (d, *J* = 10.1 Hz, 1H, −CH_2_Bn), 4.71 (ms, 3H, −CH_2_Bn), 4.58 (dd, *J* = 9.8, 4.1 Hz, 2H, −CH_2_Bn), 4.42 (d, *J* = 11.7 Hz, 2H, −CH_2_Bn), 3.96 (d, *J* = 2.8 Hz, 1H, H-4), 3.88 (t, *J* = 9.4 Hz, 1H, H-2), 3.65–3.56 (ms, 4H, H-3, H-5, H-6a/b), 2.27 (s, 3H, −CH_3_).

The intermediate **20** (0.11 g, 0.17 mmol) was dissolved in acetone/H_2_O (5:1, 12 mL). NBS (0.09 g, 0.51 mmol) was added, and the mixture was heated to 60 °C, stirred for 1 h, cooled to room temperature, and diluted with EtOAc. The layers were separated, and the organic layer was washed with saturated aqueous NaHCO_3_ (15 mL) and brine (15 mL). The organic layer was dried over Na_2_SO_4_, and the solvents were removed under reduced pressure. Column chromatography (25% EtOAc in cyclohexane) gave the hemiacetal **13** (0.075 g, 0.138 mmol, 82%) as a mixture of anomers. All analytical data were in agreement with those previously reported in the literature.^58^

^1^H NMR (400 MHz, CDCl_3_) δ 7.43–7.26 (ms, 20H), 5.30 (d, *J* = 3.2 Hz, 1H, H-1α), 4.96 (ms, 2H, −CH_2_Bn), 4.85–4.70 (ms, 6H, −CH_2_Bn), 4.65 (t, *J* = 7.5 Hz, 1H, H-1β), 4.60 (m, 1H, −C(H)HBn), 4.49 (m, 1H, −C(H)HBn), 4.40 (m, 1H, −C(H)HBn), 4.19 (t, *J* = 6.3 Hz, 1H, H-5α), 4.05 (dd, *J* = 9.4, 3.6 Hz, 1H, H-2α), 3.97 (d, *J* = 2.8 Hz, 1H, H-4α), 3.88 (d, *J* = 2.9 Hz, 1H, H-3α), 3.80 (dd, *J* = 9.7, 7.5 Hz, 1H, H-2β), 3.64–3.49 (ms, 2H, H-5β, H-3β), 3.45 (dd, *J* = 9.4, 6.2 Hz, 2H, H-6α).

^13^C NMR (101 MHz, CDCl_3_) δ 138.8 (Ar C), 138.6 (Ar C), 138.5 (Ar C), 138.4 (Ar C), 137.9 (Ar C), 128.6 (Ar CH), 128.6 (Ar CH), 128.5 (Ar CH), 128.5 (Ar CH), 128.4 (Ar CH), 128.3 (Ar CH), 128.2 (Ar CH), 128.2 (Ar CH), 127.9 (Ar CH), 127.8 (Ar CH), 127.7 (Ar CH), 97.9 (C-1β), 92.0 (C-1α), 82.3 (C-3β), 80.8 (C-2β), 78.9 (C-3α), 76.6 (C-2α), 75.2 (−CH_2_Bn), 74.9 (C-4α), 74.8 (−CH_2_Bn), 74.7 (−CH_2_Bn), 73.6 (C-5β), 73.6 (C-4β), 73.5 (−CH_2_Bn), 73.1 (−CH_2_Bn), 73.1 (−CH_2_Bn), 69.5 (C-5α), 69.3 (C-6α), 69.0 (C-6β).

### Batch Formation of Ethyl (4*S*,5*R*,6*R*)-4,5,6-Tri-*O*-benzyl-7-hydroxyhept-2-enoate (**9**)

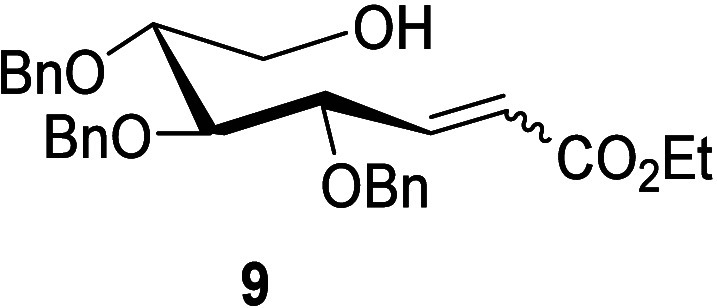



Hemiacetal **8** (0.51 g, 1.21 mmol) was dissolved in toluene (20 mL), and Ph_3_PCHCO_2_Et (1.26 g, 3.63 mmol) was added. The mixture was heated to 90 °C and stirred for 18 h, after which it was cooled to room temperature, and the solvent was removed under reduced pressure. Column chromatography (4:1 cyclohexane/EtOAc) gave **9-(E)** (0.43 g, 72%) and **9-(Z)** (0.062 g, 10%) as clear oils. All analytical data for the products were in agreement with those previously reported.^59^

**9-(E)** (major): ^1^H NMR (500 MHz, CDCl_3_) δ 7.37–7.27 (ms, 15H, Ar H), 6.97 (dd, *J* = 15.8, 5.9 Hz, 1H, H-3), 6.07 (dd, *J* = 15.8, 1.4 Hz, 1H, H-2), 4.70 (s, 2H, −CH_2_Bn), 4.62 (ms, 3H, −CH_2_Bn), 4.40 (d, *J* = 11.6 Hz, 1H, −CH_2_Bn), 4.26 (ddd, *J* = 6.1, 4.9, 1.5 Hz, 1H, H-4), 4.21 (qd, *J* = 7.2, 1.6 Hz, 2H, −C**H**_**2**_CH_3_), 3.71 (ms, 2H, H-5/H-7a), 3.62 (dd, *J* = 10.2, 4.3 Hz, 1H, H-6), 3.54 (dt, *J* = 10.0, 4.7 Hz, 1H, H-7b), 2.03 (dd, *J* = 7.0, 5.8 Hz, 1H, −OH), 1.31 (t, *J* = 7.1 Hz, 3H, −CH_2_C**H**_**3**_).

^13^C NMR (126 MHz, CDCl_3_) δ 165.9 (C=O), 144.7 (C-3), 138.1 (Ar C), 137.8 (Ar C), 137.4 (Ar C), 128.5 (Ar CH), 128.5 (Ar CH), 128.4 (Ar CH), 128.4 (Ar CH), 128.0 (Ar CH), 128.0 (Ar CH), 127.8 (Ar CH), 123.3 (C-2), 80.7 (C-5), 79.3 (C-6), 78.3 (C-4), 74.7 (−CH_2_Bn), 72.9 (−CH_2_Bn), 71.8 (−CH_2_Bn), 61.4 (C-7), 60.5 (−CO_2_**C**H_2_CH_3_), 14.2 (−CO_2_CH_2_**C**H_3_).

**9-(Z)** (minor): ^1^H NMR (500 MHz, CDCl_3_) δ 7.36–7.26 (ms, 15H, Ar H), 6.36 (dd, *J* = 11.8, 8.2 Hz, 1H, H-3), 5.80 (dd, *J* = 11.7, 1.3 Hz, 1H, H-2), 5.17 (d, *J* = 9.6 Hz, 1H, H-4), 4.76 (d, *J* = 11.5 Hz, 2H, −CH_2_Bn), 4.65–4.56 (ms, 3H, −CH_2_Bn), 4.34 (d, *J* = 11.8 Hz, 1H, −CH_2_Bn), 4.12 (q, *J* = 7.1 Hz, 2H, −C**H**_**2**_CH_3_), 3.85–3.80 (ms, 2H, H-5/H-6), 3.66 (apt td or ddd, *J* = 9.0, 7.7, 5.0 Hz, 1H, H-7a), 3.47 (m, 1H, H-7b), 2.25 (dd, *J* = 7.8, 5.5 Hz, 1H, −OH), 1.25 (t, *J* = 7.1 Hz, 3H, −CH_2_C**H**_**3**_).

^13^C NMR (126 MHz, CDCl_3_) δ 165.8 (C=O), 148.8 (C-3), 138.5 (Ar C), 138.1 (Ar C), 137.5 (Ar C), 128.6 (Ar CH), 128.4 (Ar CH), 128.4 (Ar CH), 128.2 (Ar CH), 127.9 (Ar CH), 127.9 (Ar CH), 127.7 (Ar CH), 127.6 (Ar CH), 122.0 (C-2), 81.4 (C-5), 80.3 (C-6), 75.1 (−CH_2_Bn), 73.8 (C-4), 73.2 (−CH_2_Bn), 71.5 (−CH_2_Bn), 61.6 (C-7), 60.5 (−CO_2_**C**H_2_CH_3_), 14.2 (−CO_2_CH_2_**C**H_3_).

### General Flow Procedure (a) for Synthesis of **7b**, **10**, **11**, **12**, **14**, and **16**

**Figure 1:**
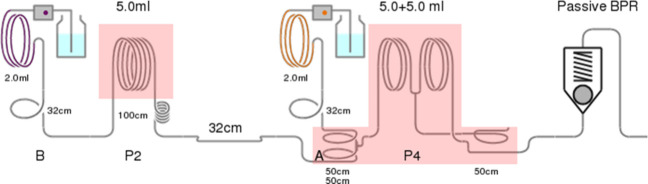
Vapourtec flow setup used in general procedure (a).

The Vapourtec R-Series system was configured as shown in Figure 1 and primed with toluene. The hemiacetal and Ph_3_PCHCO_2_Et (3–5 equiv) were premixed in toluene and injected into 2 mL sample loop B, and DBU (1.5 equiv in toluene) was injected into 2 mL sample loop A. The residence times and temperatures were set using Flow Commander software. After collection, the solvent was removed under reduced pressure, and column chromatography gave the respective products.

### Ethyl 2-(2,3,4,6-Tetra-*O*-benzyl-β-d-glucopyranosyl)ethanoate (**7b**)

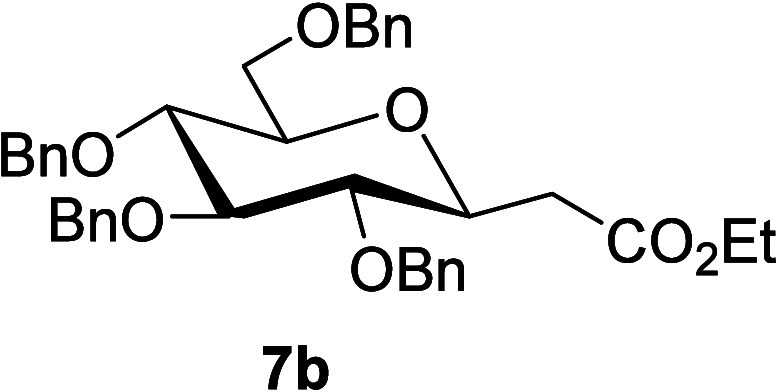



The synthesis of **7b** was carried out using general procedure (a) using hemiacetal **3** (0.09 g, 0.17 mmol) and Ph_3_PCHCO_2_Et (0.29 g, 0.83 mmol), and DBU (37 μL, 0.25 mmol). Temperatures in reactor 1 and reactor 2 were set to 200 and 145 °C, respectively, and a 16 bar pressure as set by the BPR. Residence times in both reactors were set to 40 min, and flow rates in both pumps were 0.125 mL/min. Column chromatography (7:1 to 4:1 cyclohexane/EtOAc) gave the Michael addition product **7b** (0.068 g, 0.11 mmol, 67%) as a clear gel. The intermediate Wittig product **11-E** (0.008 g, 7%) was also isolated. All analytical data for **7b** were in agreement with those previously reported.^19,60^

^1^H NMR (500 MHz, CDCl_3_) δ 7.35–7.26 (ms, 18H, Ar H), 7.17 (m, 2H, Ar H), 4.97–4.81 (ms, 4H, −CH_2_Bn), 4.68–4.49 (ms, 4H, −CH_2_Bn), 4.09 (ms, 2H, −CO_2_C**H**_**2**_CH_3_), 3.81–3.61 (ms, 5H, H-1, H-3, H-4, H-6a/b), 3.47 (dt, *J* = 9.6, 3.0 Hz, 1H, H-5), 3.38 (t, *J* = 9.2 Hz, 1H, H-2), 2.74 (m, 1H, −CH(**H**)CO_2_Et), 2.48 (dd, *J* = 15.3, 8.3 Hz, 1H, −CH(**H**)CO_2_Et), 1.20 (t, *J* = 7.1 Hz, 3H, −CO_2_CH_2_C**H**_**3**_).

^13^C NMR (126 MHz, CDCl_3_) δ 171.0 (C=O), 138.5 (Ar C), 138.2 (Ar C), 138.2 (Ar C), 138.0 (Ar C), 128.4 (Ar CH), 128.4 (Ar CH), 128.4 (Ar CH), 128.3 (Ar CH), 127.9 (Ar CH), 127.9 (Ar CH), 127.8 (Ar CH), 127.8 (Ar CH), 127.7 (Ar CH), 127.6 (Ar CH), 127.6 (Ar CH), 87.2 (C-1/C-3/C-4), 81.3 (C-2), 79.2 (C-5), 78.5 (C-1/C-3/C-4), 76.0 (C-1/C-3/C-4), 75.5 (−CH_2_Bn), 75.0 (−CH_2_Bn), 75.0 (−CH_2_Bn), 73.4 (−CH_2_Bn), 68.7 (C-6), 60.5 (−**C**H_2_CH_3_), 37.6 (−CH_2_CO_2_Et), 14.2 (−CH_2_**C**H_3_).

### (*E*)-(4*S*,5*R*,6*R*,7*R*)-4,5,6,8-Tetra-*O*-benzyl-7-hydroxyoct-2-enoic Acid Ethyl Ester (**11-(E)**)

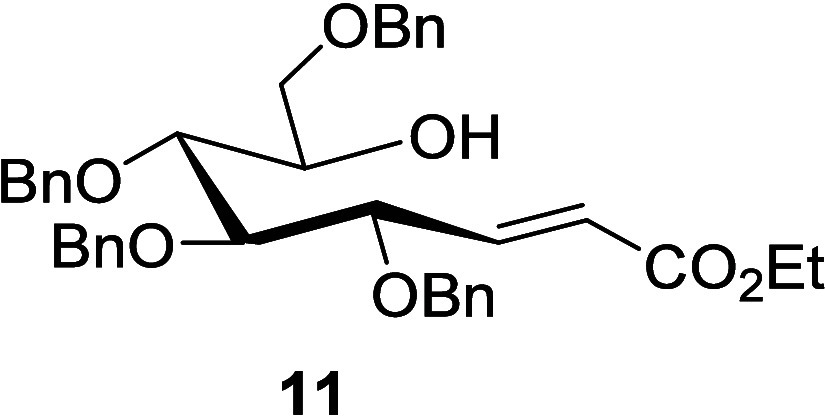



^1^H NMR (500 MHz, CDCl_3_) δ 7.41–7.26 (ms, 20H), 7.13 (d, *J* = 15.7 Hz, 1H, H-2), 6.13 (d, *J* = 15.7 Hz, 1H, H-3), 5.47 (d, *J* = 9.5 Hz, 1H, H-4), 4.70 (d, *J* = 3.4 Hz, 2H, −CH_2_Bn), 4.47–4.40 (ms, 4H, −CH_2_Bn), 4.41 (m, 1H, H-8a), 4.38 (dd, *J* = 9.6, 5.7 Hz, 1H, H-5), 4.19 (ms, 3H, H-8b, −C**H**_**2**_CH_3_), 3.83 (m, 1H, H-7), 3.47 (ms, 3H, H-6, −CH_2_Bn), 1.28 (t, *J* = 7.1 Hz, 3H, −CH_2_C**H**_**3**_).

^13^C NMR (101 MHz, CDCl_3_) δ 166.7 (C=O), 139.7 (C-2), 138.1 (Ar C), 138.0 (Ar C), 136.7 (Ar C), 128.7 (Ar CH), 128.7 (Ar CH), 128.5 (Ar CH), 128.4 (Ar CH), 128.3 (Ar CH), 128.2 (Ar CH), 127.9 (Ar CH), 127.8 (Ar CH), 127.8 (Ar CH), 123.9 (C-4), 120.0 (C-3), 74.4 (−CH_2_Bn), 73.6 (−CH_2_Bn), 73.5 (−CH_2_Bn), 72.3 (C-7), 70.8 (C-6), 70.7 (C-8), 60.8 (−**C**H_2_CH_3_), 14.4 (−CH_2_**C**H_3_).

### Ethyl 2-(2,3,4-Tri-*O*-benzyl-β-d-xylopyranosyl)ethanoate (**10**) and Ethyl 2-(2,3,4-Tri-*O*-benzyl-α-d-xylopyranosyl)ethanoate (**12**)

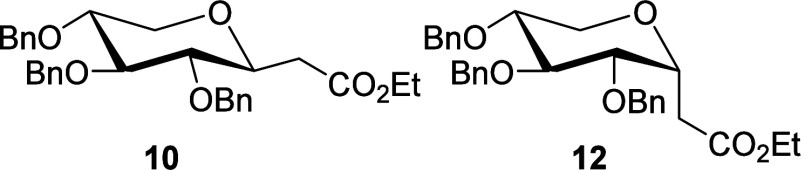



The synthesis of **10** and **12** was carried out using general procedure (a) using compound **8** (0.09 g, 0.214 mmol) and Ph_3_PCHCO_2_Et (0.37 g, 1.07 mmol), and DBU (48 μL, 0.32 mmol). Temperatures in reactor 1 and reactor 2 were set to 180 and 130 °C, respectively, and an 8 bar BPR was used. Residence times in both reactors were set to 45 min, and flow rates in both pumps were 0.11 mL/min. Column chromatography (7:1 to 5:1 cyclohexane/EtOAc) gave the Michael addition products **10** (0.058 g, 0.118 mmol, 55%) and **12** (0.016 g, 0.033 mmol, 15%) and the Wittig product **9-(E)** (0.010 g, 0.0218 mmol, 10%).

β product **10** (major): ^1^H NMR (500 MHz, CDCl_3_) δ 7.35–7.26 (ms, 15H), 4.99 (d, *J* = 10.9 Hz, 1H, −CH_2_Bn), 4.93 (d, *J* = 11.1 Hz, 1H, −CH_2_Bn), 4.84 (d, *J* = 10.9 Hz, 1H, −CH_2_Bn), 4.71 (d, *J* = 11.6 Hz, 1H, −CH_2_Bn), 4.62 (dd, *J* = 11.4, 5.5 Hz, 2H, −CH_2_Bn), 4.11 (q, *J* = 7.1 Hz, 2H, −CO_2_C**H**_**2**_CH_3_), 3.96 (dd, *J* = 11.4, 4.9 Hz, 1H, H-5a), 3.64 (ms, 3H, H-1/H-3/H-4, overlapping signals), 3.28 (t, *J* = 8.7 Hz, 1H, H-2), 3.21 (dd, *J* = 11.4, 10.0 Hz, 1H, H-5b), 2.74 (dd, *J* = 15.2, 3.3 Hz, 1H, −CH(**H**)CO_2_Et), 2.36 (m, 1H, −CH(**H**)CO_2_Et), 1.22 (t, *J* = 7.1 Hz, 3H, −CO_2_CH_2_C**H**_**3**_).

^13^C NMR (126 MHz, CDCl_3_) δ 171.0 (C=O), 138.6 (Ar C), 138.1 (Ar C), 138.1 (Ar C), 128.5 (Ar CH), 128.4 (Ar CH), 128.4 (Ar CH), 127.9 (Ar CH), 127.9 (Ar CH), 127.8 (Ar CH), 127.8 (Ar CH), 127.8 (Ar CH), 127.7 (Ar CH), 86.2 (C-3), 80.9 (C-2), 78.7 (C-4), 76.5 (C-1), 75.5 (−**C**H_2_Bn), 75.1 (−**C**H_2_Bn), 73.3 (−**C**H_2_Bn), 68.1 (C-5), 60.6 (−CO_2_**C**H_2_CH_3_), 37.7 (**C**H_2_CO_2_Et), 14.1 (−CO_2_CH_2_**C**H_3_).

ESI-QTOF-HRMS [M + H]^+^: calcd 491.2434; found 491.2446 (error 2.4 ppm).

FT-IR (neat): 3456, 2980, 2873, 1726, 1603, 1495, 1453, 1368, 1317, 1263, 1177, 1070, 1025, 910, 738 cm^–1^.

α product **12** (minor): ^1^H NMR (500 MHz, CDCl_3_) δ 7.36–7.26 (ms, 15H, Ar H), 4.66 (d, *J* = 11.8 Hz, 1H, −CH_2_Bn), 4.61 (d, *J* = 5.1 Hz, 4H, −CH_2_Bn), 4.52 (d, *J* = 11.8 Hz, 1H, −CH_2_Bn), 4.32 (ddd, *J* = 8.8, 5.4, 3.6 Hz, 1H, H-1), 4.12 (m, 2H, −C**H**_**2**_CH_3_), 3.78 (ms, 2H, H-5a and H-5b), 3.70 (t, *J* = 5.2 Hz, 1H, H-3), 3.52 (dd, *J* = 5.2, 3.7 Hz, 1H, H-2), 3.44 (q, *J* = 5.1 Hz, 1H, H-4), 2.77 (dd, *J* = 15.9, 8.6 Hz, 1H, −CH(**H**)CO_2_Et), 2.64 (dd, *J* = 15.9, 5.5 Hz, 1H, −CH(**H**)CO_2_Et), 1.24 (t, *J* = 7.1 Hz, 3H, −CH_2_C**H**_**3**_).

^13^C NMR (126 MHz, CDCl_3_) δ 171.4 (C=O), 138.3 (Ar C), 138.2 (Ar C), 138.0 (Ar C), 128.4 (Ar CH), 128.4 (Ar CH), 128.4 (Ar CH), 128.2 (Ar CH), 127.9 (Ar CH), 127.8 (Ar CH), 127.7 (Ar CH), 75.9 (C-2), 74.9 (C-3), 74.6 (C-4), 73.4 (−CH_2_Bn), 72.6 (−CH_2_Bn), 72.1 (−CH_2_Bn), 72.0 (C-1), 64.8 (C-5), 60.5 (−**C**H_2_CH_3_), 34.4 (−**C**H_2_CO_2_Et), 14.2 (−CH_2_**C**H_3_).

ESI-QTOF-HRMS [M + Na]^+^: calcd 513.2253; found 513.2258 (error 0.97 ppm).

FT-IR (neat): 3031, 2871, 1732, 1497, 1454, 1367, 1273, 1254, 1182, 1073, 1027, 736 cm^–1^.

### Ethyl 2-(2,3,4,6-Tetra-*O*-benzyl-d-galactopyranosyl)ethanoate (**14**)

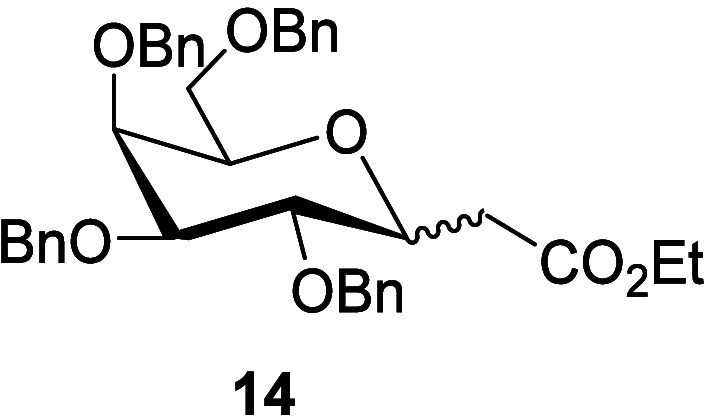



The synthesis of **14** was carried out using general procedure (a) using premixed compound **13** (0.095 g, 0.176 mmol) and Ph_3_PCHCO_2_Et (0.31 g, 0.88 mmol) and DBU (39 μL, 0.26 mmol). Temperatures in reactor 1 and reactor 2 were set to 180 and 130 °C, respectively, and an 8 bar BPR was used. Residence times in both reactors were set to 45 min, and flow rates in both pumps were 0.11 mL/min. Column chromatography (7:1 to 5:1 cyclohexane/EtOAc) gave the Michael addition product **14** (0.065 g, 0.106 mmol, 60%) as a mixture of anomers. All analytical data were in agreement with those previously reported.^61^

^1^H NMR (400 MHz, CDCl_3_) δ 7.39–7.26 (ms, Ar H), 4.96 (dd, *J* = 18.7, 11.3 Hz, −CH_2_Bn), 4.79–4.39 (ms, −CH_2_Bn, H-1α, overlapping signals), 4.05 (ms, −CO_2_C**H**_**2**_CH_3_ α, −CO_2_C**H**_**2**_CH_3_ β, H-2β, H-4α, overlapping signals), 3.93 (m, 1H, H-2α), 3.80–3.53 (ms, H-1β, H-3β, H-4β, H-5β, H-3α, H-5α, H-6a/b α, H-6a/b β), 2.78 (dd, *J* = 15.4, 3.3 Hz, 1H, −CH(**H**)CO_2_Et β), 2.65 (apt d, *J* = 7.1 Hz, −C**H**(H)CO_2_Et α), 2.49 (dd, *J* = 15.4, 8.1 Hz, 1H, −CH(**H**)CO_2_Et β), 1.18 (t, *J* = 7.1 Hz, CO_2_CH_2_C**H**_**3**_ α), 1.17 (t, *J* = 7.1 Hz, −CO_2_CH_2_C**H**_**3**_ β).

^13^C NMR (101 MHz, CDCl_3_) δ 171.4 (C=O α), 171.3 (C=O β), 138.8 (Ar C), 138.5 (Ar C), 138.5 (Ar C), 138.4 (Ar C), 138.4 (Ar C), 138.3 (Ar C), 138.2 (Ar C), 138.0 (Ar C), 128.5 (Ar CH), 128.5 (Ar CH), 128.4 (Ar CH), 128.3 (Ar CH), 128.1 (Ar CH), 128.0 (Ar CH), 127.9 (Ar CH), 127.8 (Ar CH), 127.7 (Ar CH), 127.6 (Ar CH), 84.8 (C-5β), 78.2 (C-4β), 77.3 (C-3β), 76.5 (C-1β), 76.0 (C-2α), 75.3 (−CH_2_Bn), 74.7 (−CH_2_Bn), 74.1 (C-4α), 73.8 (C-2β), 73.5 (−CH_2_Bn), 73.3 (−CH_2_Bn), 72.9 (−CH_2_Bn), 72.2 (−CH_2_Bn), 68.7 (C-6β), 67.6 (C-6α), 60.6 (−**C**H_2_CH_3_ α), 60.5 (−**C**H_2_CH_3_ β), 38.0 (−**C**H_2_CO_2_Et β), 29.8 (−**C**H_2_CO_2_Et α), 14.2 (−CH_2_**C**H_3_ α), 14.2 (−CH_2_**C**H_3_ β).

### Ethyl 2-(2,3,4,6-Tetra-*O*-benzyl-α-d-mannopyranosyl)ethanoate (**16**)

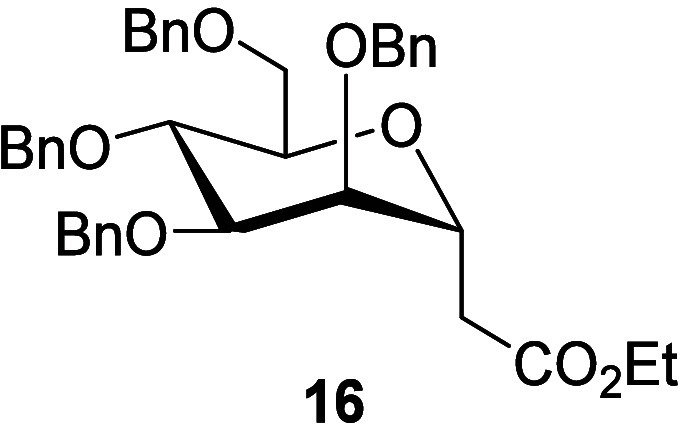



The synthesis of **16** was carried out using general procedure (a) using hemiacetal **15** (0.108 g, 0.199 mmol) and Ph_3_PCHCO_2_Et (0.35 g, 0.99 mmol), and DBU (44 μL, 0.29 mmol). Temperatures in reactor 1 and reactor 2 were set to 180 and 130 °C, respectively, and an 8 bar BPR was used. Residence times in both reactors were set to 45 min, and flow rates in both pumps were 0.11 mL/min. Column chromatography (7:1 to 5:1 cyclohexane/EtOAc) gave the Michael addition product **16** (0.032 g, 0.052 mmol, 26%) as a gel. All analytical data were in agreement with those previously reported.^21^

^1^H NMR (500 MHz, CDCl_3_) δ 7.35–7.21 (ms, 20H), 4.62 (d, *J* = 11.6 Hz, 1H, −CH_2_Bn), 4.55 (ms, 5H, −CH_2_Bn), 4.52–4.49 (ms, 2H, −CH_2_Bn, H-1), 4.11 (q, *J* = 7.2 Hz, 2H, −C**H**_**2**_CH_3_), 3.92 (dd, *J* = 9.9, 4.9 Hz, 1H, H-5), 3.88 (t, *J* = 5.9, 5.1 Hz, 1H, H-4), 3.80 (ms, 3H, H-3, H-6a/H-6b), 3.66 (dd, *J* = 6.4, 2.9 Hz, 1H, H-2), 2.67 (dd, *J* = 15.1, 5.3 Hz, 1H, −CH(**H**)CO_2_Et), 2.55 (dd, *J* = 15.2, 8.4 Hz, 1H, −CH(**H**)CO_2_Et), 1.21 (t, *J* = 7.1 Hz, 3H, −CH_2_C**H**_**3**_).

^13^C NMR (126 MHz, CDCl_3_) δ 171.0 (C=O), 138.4 (Ar C), 138.2 (Ar C), 138.1 (Ar C), 138.0 (Ar C), 128.4 (Ar CH), 128.4 (Ar CH), 128.4 (Ar CH), 128.3 (Ar CH), 128.0 (Ar CH), 127.9 (Ar CH), 127.9 (Ar CH), 127.7 (Ar CH), 127.5 (Ar CH), 75.4 (C-2), 74.4 (C-4), 74.3 (C-5), 73.3 (−CH_2_Bn), 73.1 (−CH_2_Bn), 72.2 (−CH_2_Bn), 71.3 (−CH_2_Bn), 68.8 (C-1), 60.6 (−**C**H_2_CH_3_), 36.6 (−**C**H_2_CO_2_Et), 14.2 (−CH_2_**C**H_3_).

### 2-(2,3,4,6-Tetra-*O*-benzyl-β-d-glucopyranosyl)ethanol (**17**) and 2-(2,3,4,6-Tetra-*O*-benzyl-α-d-glucopyranosyl)ethanol (**18**)

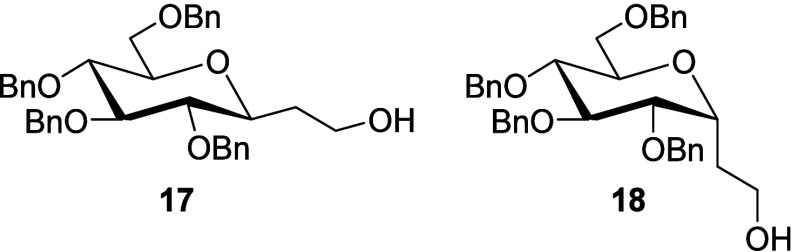



Compound **7b** (0.08 g, 0.13 mmol) was dissolved in DCM (5 mL), and the solution was cooled to −5 °C. DIBAL-H (0.17 mL, 0.2 mmol, 1.2 M in toluene) was charged under N_2_ atmosphere. The reaction mixture was warmed to room temperature after 10 min. The mixture was stirred for 4 h and quenched with EtOAc (10 mL). Saturated aqueous Rochelle salt (10 mL) was added, and the mixture was stirred for 1 h. The mixture was diluted with EtOAc, and the layers were separated. The organic layer was washed with H_2_O (10 mL) and brine (10 mL), and the organic layer was dried over Na_2_SO_4_. The solvent was removed under reduced pressure, and column chromatography (15% to 25% EtOAc in cyclohexane) gave **17** (0.042 g, 0.074 mmol, 56%) and **18** (0.014 g, 0.024 mmol, 19%) as clear gels. All analytical data were in agreement with those previously reported.^62^

β product **17** (major): ^1^H NMR (500 MHz, CDCl_3_) δ 7.38–7.17 (ms, 20H, Ar H), 4.93 (ms, 3H, −CH_2_Bn), 4.85 (d, *J* = 10.8 Hz, 1H, −CH_2_Bn), 4.66 (d, *J* = 11.0 Hz, 1H, −CH_2_Bn), 4.60–4.51 (ms, 3H, −CH_2_Bn), 3.80 (t, *J* = 5.1 Hz, 2H, −CH_2_C**H**_**2**_OH), 3.72 (t, *J* = 8.8 Hz, 1H, H-3), 3.69 (dd, *J* = 9.1, 2.1 Hz, 1H, H-6a), 3.61 (dd, *J* = 10.6, 5.1 Hz, 1H, H-6b), 3.59 (t, *J* = 9.0 Hz, 1H, H-2), 3.51 (ms, 2H, H-1, H-5), 3.36 (t, *J* = 9.2 Hz, 1H, H-4), 2.08 (dq, *J* = 14.7, 2.9 Hz, 1H, −CH(**H**)CH_2_OH), 1.77 (m, 1H, −CH(**H**)CH_2_OH).

^13^C NMR (126 MHz, CDCl_3_) δ 138.5 (Ar C), 138.0 (Ar C), 137.9 (Ar C), 137.9 (Ar C), 128.5 (Ar CH), 128.5 (Ar CH), 128.5 (Ar CH), 128.5 (Ar CH), 128.0 (Ar CH), 128.0 (Ar CH), 127.9 (Ar CH), 127.9 (Ar CH), 127.8 (Ar CH), 127.7 (Ar CH), 127.7 (Ar CH), 127.7 (Ar CH), 87.0 (C-3), 81.8 (C-4), 79.8 (C-1/C-5), 78.7 (C-1/C-5), 78.5 (C-2), 75.6 (−CH_2_Bn), 75.3 (−CH_2_Bn), 75.1 (−CH_2_Bn), 73.5 (−CH_2_Bn), 69.1 (C-6), 61.5 (−CH_2_**C**H_2_OH), 33.8 (−**C**H_2_CH_2_OH).

α product **18** (minor): ^1^H NMR (500 MHz, CDCl_3_) δ 7.37–7.28 (ms, 18H, Ar H), 7.15 (ms, 2H, Ar H), 4.93 (m, 1H, −CH_2_Bn), 4.82 (ms, 2H, −CH_2_Bn), 4.73 (d, *J* = 11.6 Hz, 1H, −CH_2_Bn), 4.64 (d, *J* = 11.6 Hz, 1H, −CH_2_Bn), 4.58 (d, *J* = 12.3 Hz, 1H, −CH_2_Bn), 4.50 (m, 2H, −CH_2_Bn), 4.23 (ddd, *J* = 10.5, 5.8, 3.7 Hz, 1H, H-1), 3.80 (ms, 4H, H-3, H-5, −CH_2_C**H**_**2**_OH), 3.73 (dd, *J* = 9.4, 5.8 Hz, 1H, H-2), 3.65 (dd, *J* = 10.3, 2.2 Hz, 1H, H-6a), 3.59 (dd, *J* = 10.1, 5.5 Hz, 1H, H-6b), 3.51 (dd, *J* = 9.8, 8.5 Hz, 1H, H-4), 2.07 (ddt, *J* = 15.0, 10.1, 5.2 Hz, 1H, −CH(**H**)CH_2_OH), 1.90 (ddt, *J* = 15.1, 5.5, 3.8 Hz, 1H, −CH(**H**)CH_2_OH).

^13^C NMR (126 MHz, CDCl_3_) δ 138.6 (Ar C), 138.1 (Ar C), 138.0 (Ar C), 137.8 (Ar C), 128.5 (Ar CH), 128.4 (Ar CH), 128.4 (Ar CH), 128.0 (Ar CH), 128.0 (Ar CH), 127.9 (Ar CH), 127.9 (Ar CH), 127.8 (Ar CH), 127.7 (Ar CH), 127.7 (Ar CH), 82.3 (C-3), 79.7 (C-2), 78.3 (C-4), 75.5 (−CH_2_Bn), 75.0 (−CH_2_Bn), 74.1 (C-1), 73.5 (−CH_2_Bn), 73.3 (−CH_2_Bn), 71.5 (C-5), 69.3 (C-6), 61.3 (−CH_2_**C**H_2_OH), 27.8 (−**C**H_2_CH_2_OH).

### 2-(2,3,4-Tri-*O*-benzyl-β-d-xylopyranosyl)ethanol (**19**)

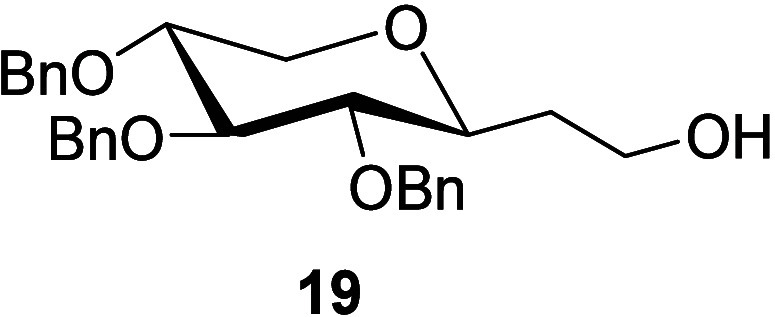



Compound **10** (0.05 g, 0.10 mmol) was dissolved in DCM (4 mL), and the solution was cooled to −5 °C. DIBAL-H (0.13 mL, 0.15 mmol, 1.2 M in toluene) was charged under N_2_ atmosphere. The reaction mixture was warmed to room temperature after 10 min. The mixture was stirred for 4 h and quenched with EtOAc (10 mL). Saturated aqueous Rochelle salt (10 mL) was added, and the mixture was stirred for 1 h. The mixture was diluted with EtOAc, and the layers were separated. The organic layer was washed with H_2_O (10 mL) and brine (10 mL), and the organic layer was dried over Na_2_SO_4_. The solvent was removed under reduced pressure, and column chromatography (25% EtOAc in cyclohexane) gave **19** (0.031 g, 0.069 mmol, 68%) as a clear gel.

^1^H NMR (500 MHz, CDCl_3_) δ 7.38–7.28 (ms, 15H, Ar H), 5.00 (d, *J* = 10.9 Hz, 1H, −CH_2_Bn), 4.93 (d, *J* = 10.9 Hz, 1H, −CH_2_Bn), 4.86 (d, *J* = 11.0 Hz, 1H, −CH_2_Bn), 4.74 (d, *J* = 11.6 Hz, 1H, −CH_2_Bn), 4.64 (apt t, *J* = 11.1 Hz, 2H, −CH_2_Bn), 3.98 (dd, *J* = 11.5, 4.8 Hz, 1H, H-5a), 3.75 (t, *J* = 5.6 Hz, 2H, −CH_2_**H**_**2**_OH), 3.63 (ms, 2H, H-3, H-4, overlapping signals), 3.42 (td, *J* = 9.3, 2.9 Hz, 1H, H-1), 3.26 (t, *J* = 9.0 Hz, 1H, H-2), 3.21 (t, *J* = 10.3 Hz, 1H, H-5b), 2.07 (dtd, *J* = 14.5, 5.2, 2.8 Hz, 1H, −CH(**H**)CH_2_OH), 1.68 (ddt, *J* = 14.6, 9.0, 5.5 Hz, 1H, −CH(**H**)CH_2_OH).

^13^C NMR (126 MHz, CDCl_3_) δ 138.6 (Ar C), 138.1 (Ar C), 138.0 (Ar C), 128.5 (Ar CH), 128.5 (Ar CH), 128.4 (Ar CH), 128.0 (Ar CH), 127.9 (Ar CH), 127.9 (Ar CH), 127.8 (Ar CH), 127.7 (Ar CH), 86.1 (C-3/C-4), 81.5 (C-2), 80.1 (C-1), 78.6 (C-3/C-4), 75.6 (−CH_2_Bn), 75.4 (−CH_2_Bn), 73.3 (−CH_2_Bn), 68.0 (C-5), 61.2 (−CH_2_**C**H_2_OH), 34.1 (−**C**H_2_CH_2_OH).

ESI-QTOF-HRMS [M + H]^+^: calcd 449.2328; found 449.2319 (error −2.0 ppm).

FT-IR (neat): 3317, 3031, 2901, 2858, 1497, 1453, 1357, 1261, 1215, 1073, 1038, 937, 901, 800, 732 cm^–1^.

## Supporting Information Available

^1^H NMR spectra of compounds **7b**, **9-(E)**, **9-(Z)**, **10**–**12**, **14**, and **16**–**19** and ^13^C NMR spectra of **10**, **12**, and **19** and results from ^1^H NMR spectroscopic analysis of crude reaction products obtained from batch and flow reactions of **3** and **8** (PDF)
