# Supplementary material for: Flow Chemistry for Synthesis of 2-(C-Glycosyl)acetates from Pyranoses via Tandem Wittig and Michael Reactions
Source: Org Process Res Dev. 2024 Feb 28;28(5):1848–59. doi: 10.1021/acs.oprd.3c00414 (PMC11110061; doi:10.1021/acs.oprd.3c00414)
Supplement: Supplementary file 1 — op3c00414_si_001.pdf [file op3c00414_si_001.pdf]

Supporting Information

Flow Chemistry for Synthesis of 2-(C-Glycosyl)acetates from Pyranoses via Tandem Wittig and Michael Reactions

Jack J. Bennett[a] and Paul V. Murphy[a,b]*

[a]School of Biological and Chemical Sciences, University of Galway, University Road, Galway, H91 TK33, Ireland.

[b]SSPC – SFI Research Centre for Pharmaceuticals, University of Galway, University Road, Galway, H91 TK33, Ireland

*Email: paul.v.murphy@universityofgalway.ie

Supporting Information Content

NMR spectra	Pages S2–S8
Table S1	Page S9

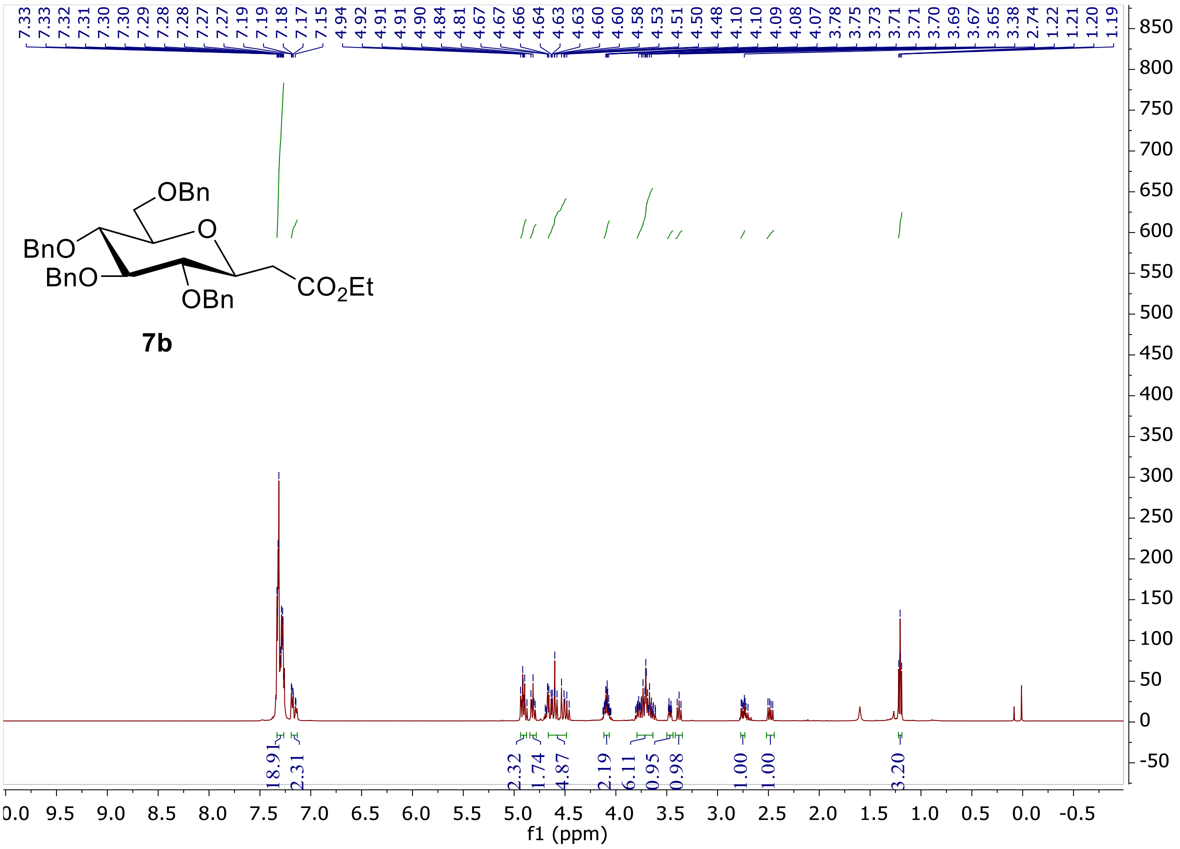

Figure S1. ^{1}H NMR spectrum of **7b**

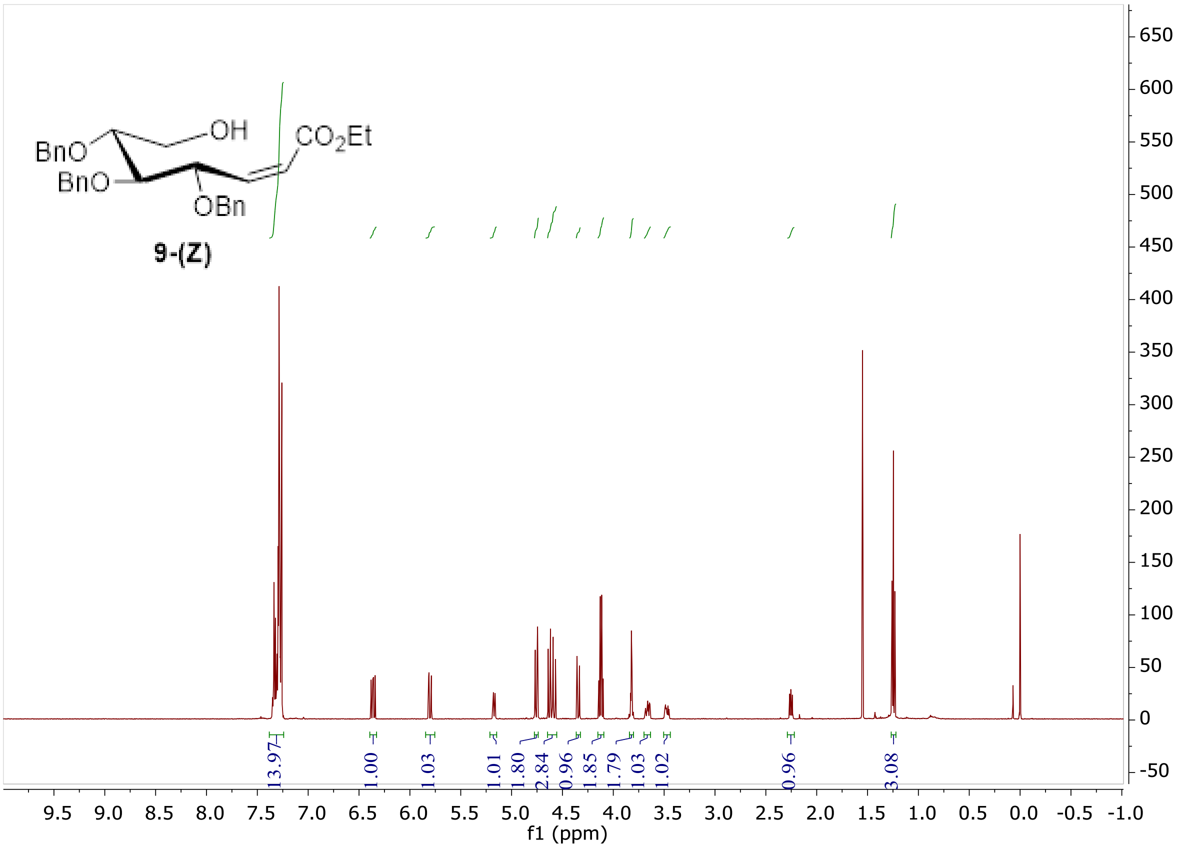

Figure S2. ^{1}H NMR spectrum of **9-(Z)**

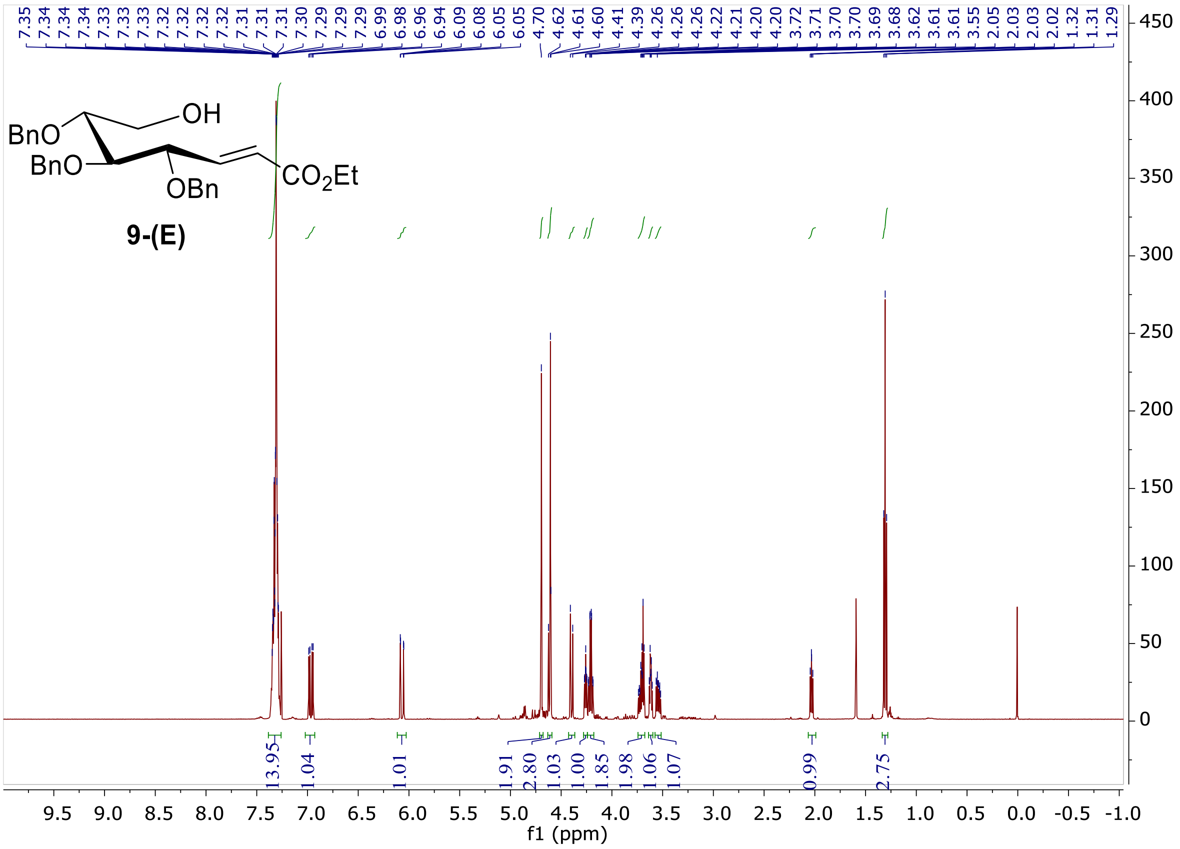

Figure S3. ^{1}H NMR spectrum of **9-(E)**

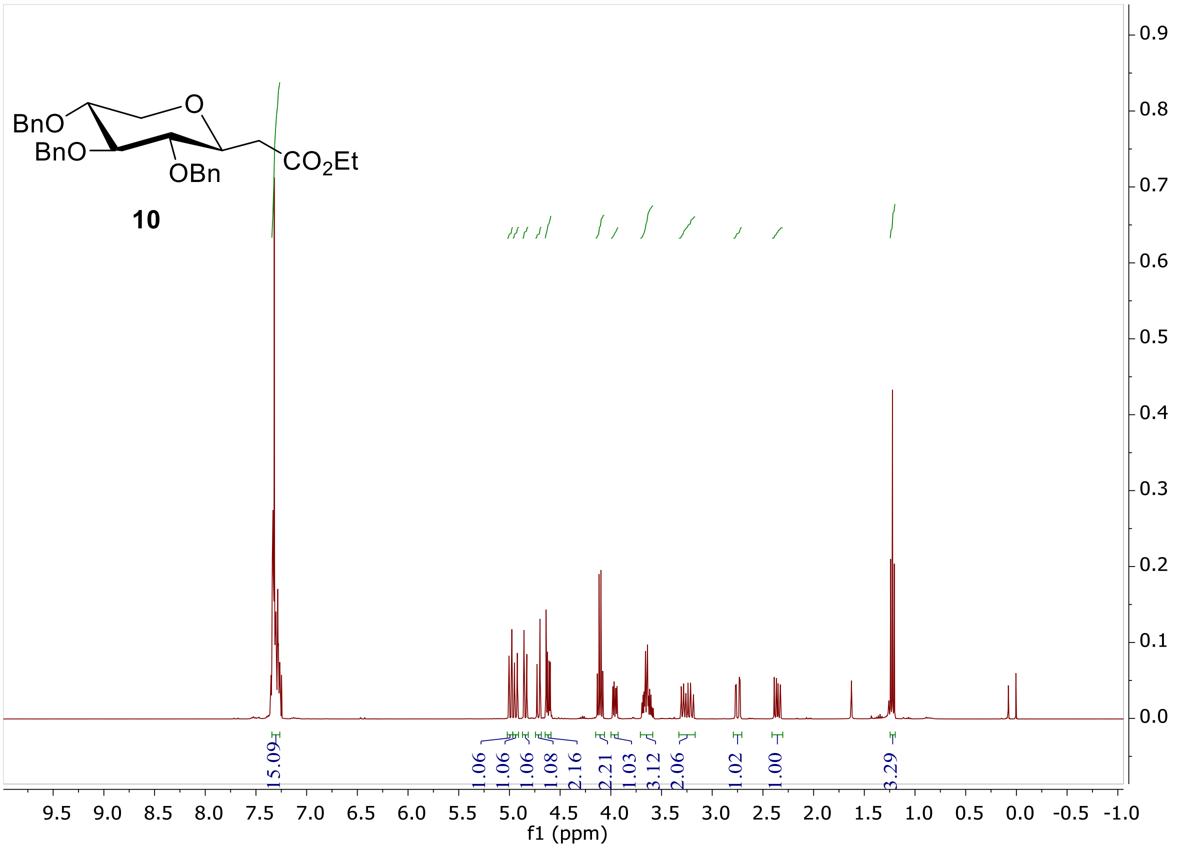

Figure S4. ^{1}H NMR spectrum of **10**

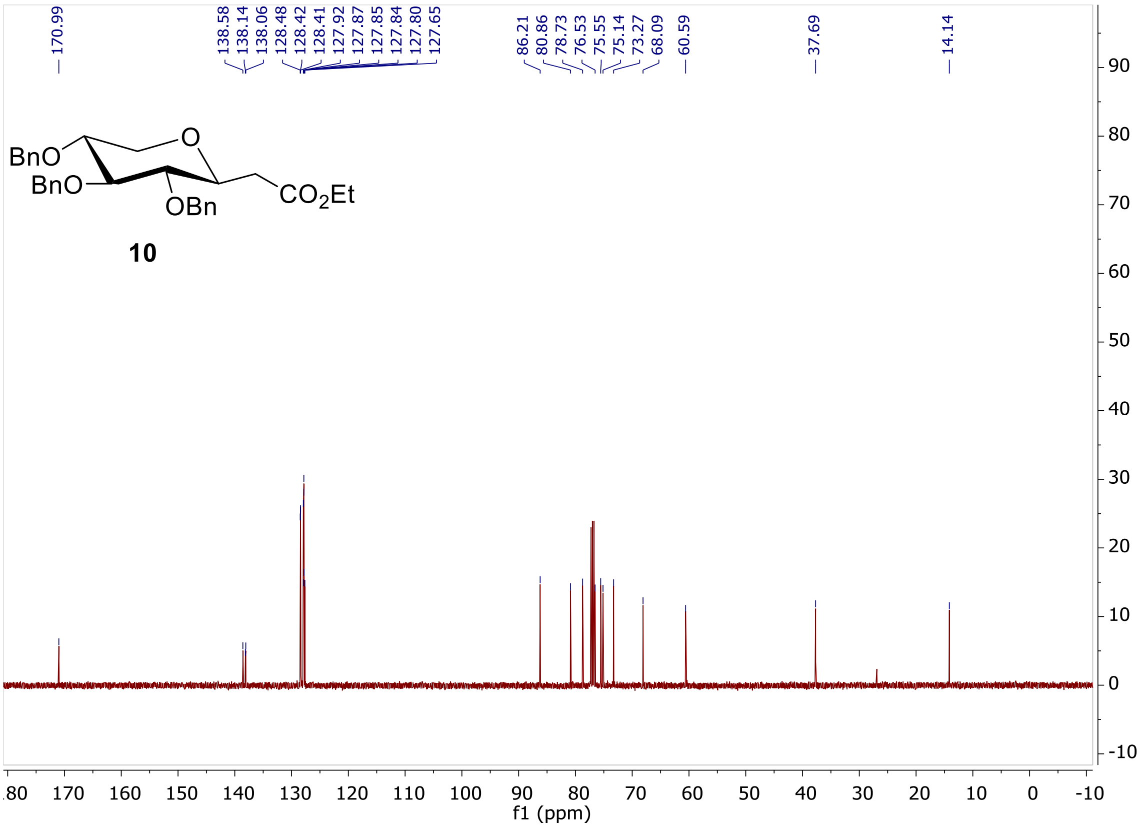

Figure S5. ^{13}C NMR spectrum of **10**

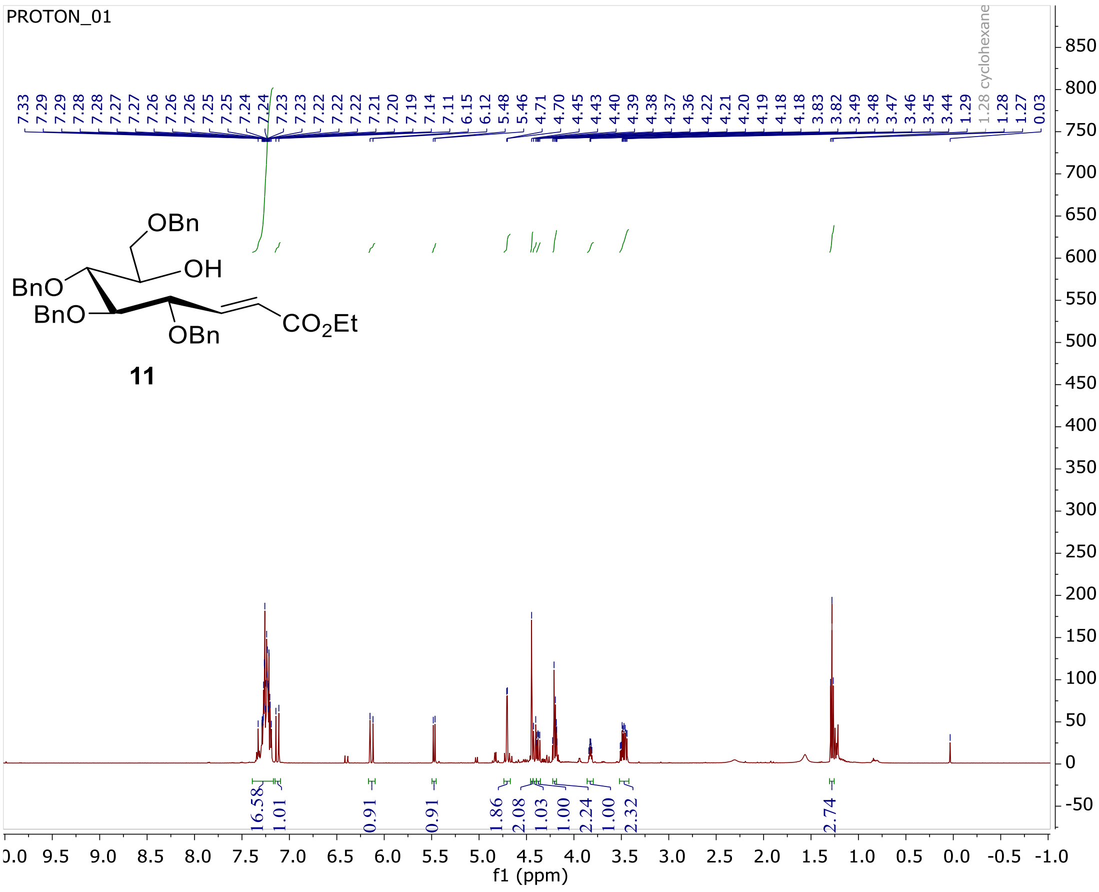

Figure S6. ^{1}H NMR spectrum of **11**

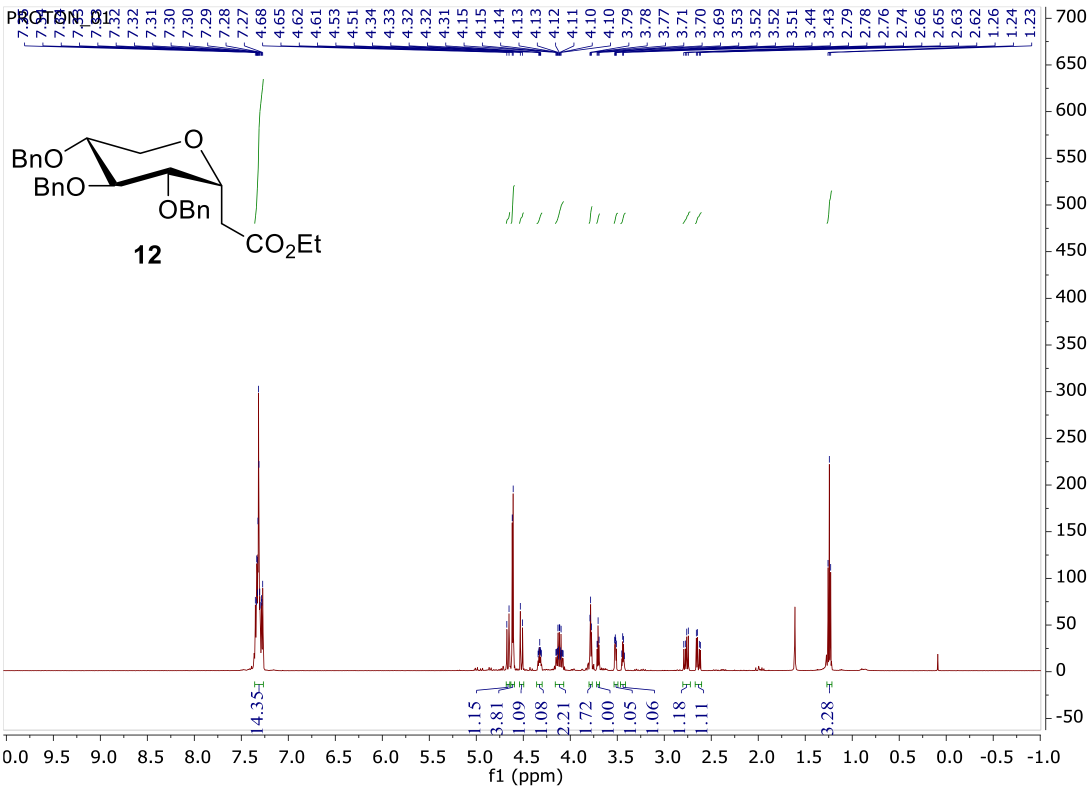

Figure S7. ^{1}H NMR spectrum of **12**

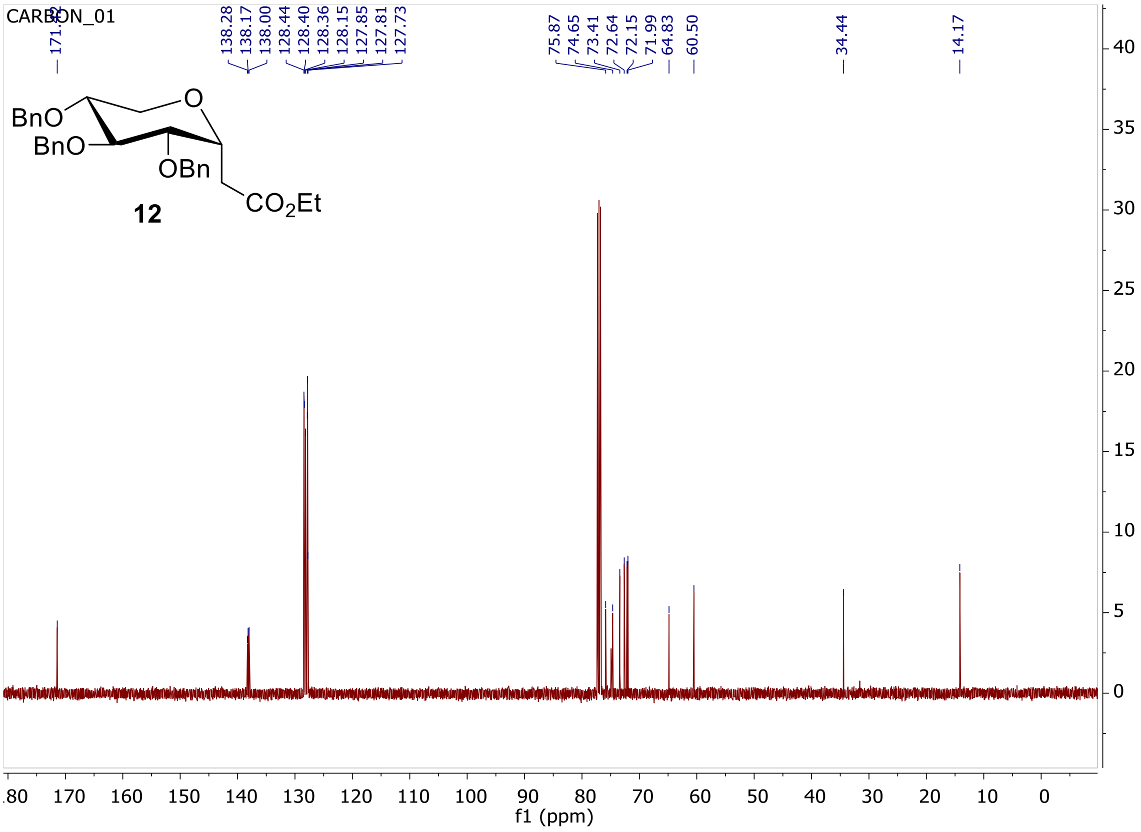

Figure S8. ^{13}C NMR spectrum of **12**

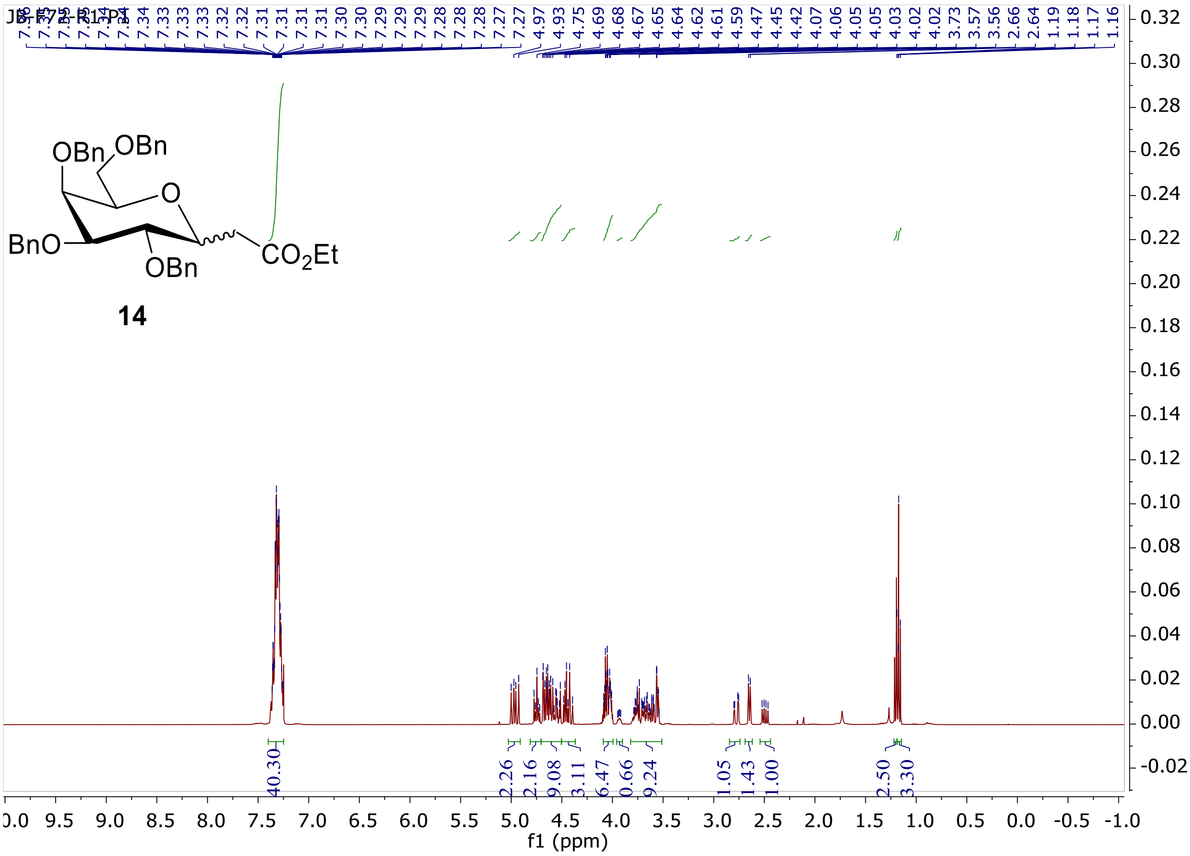

Figure S9. ^{1}H NMR spectrum of **14**

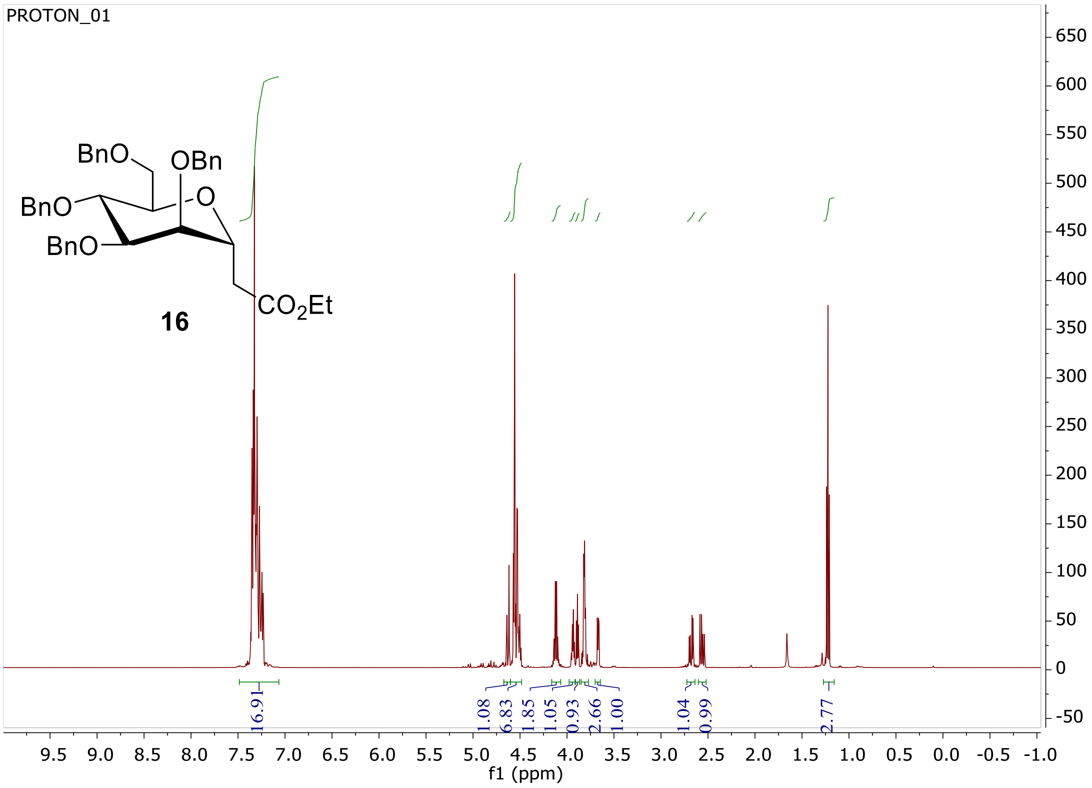

Figure S10. ^{1}H NMR spectrum of **16**

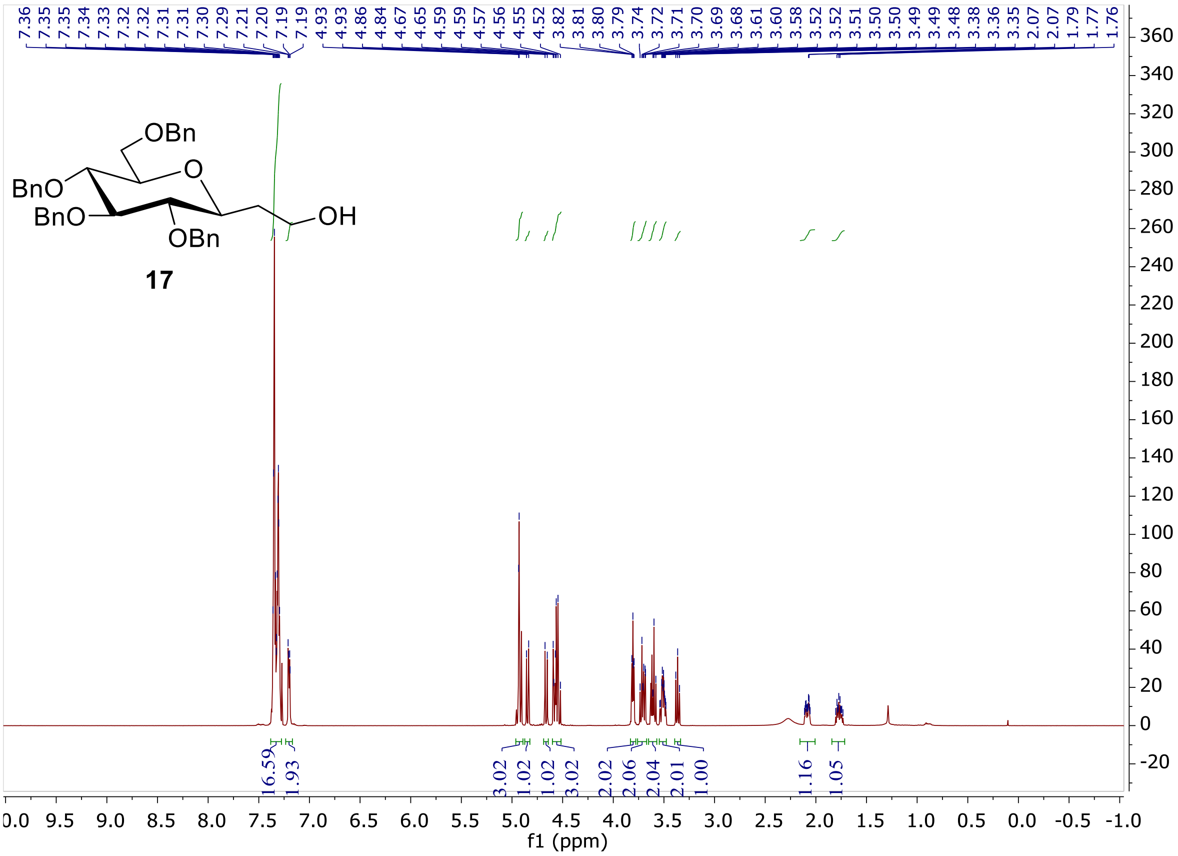

Figure S11. ^{1}H NMR spectrum of **17**

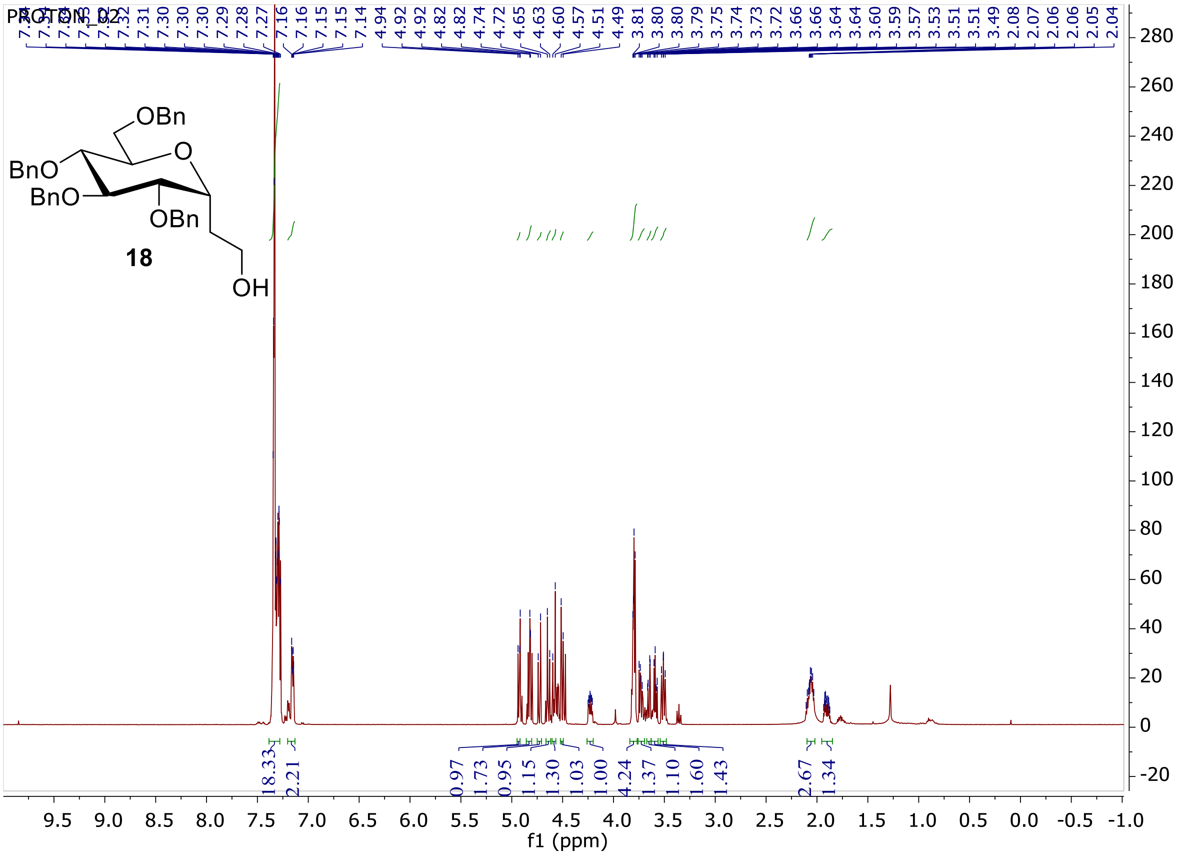

Figure S12. ^{1}H NMR spectrum of **18**

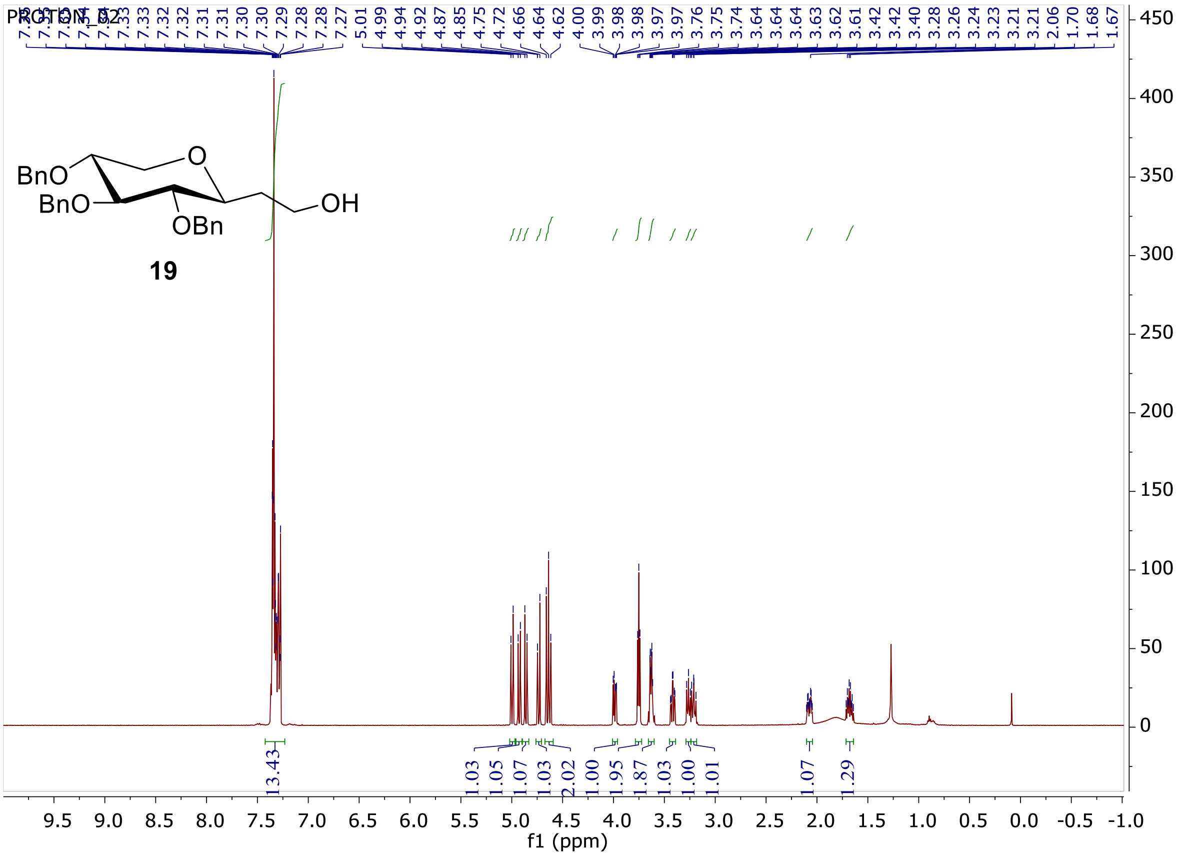

Figure S13. ^{1}H NMR spectrum of **19**

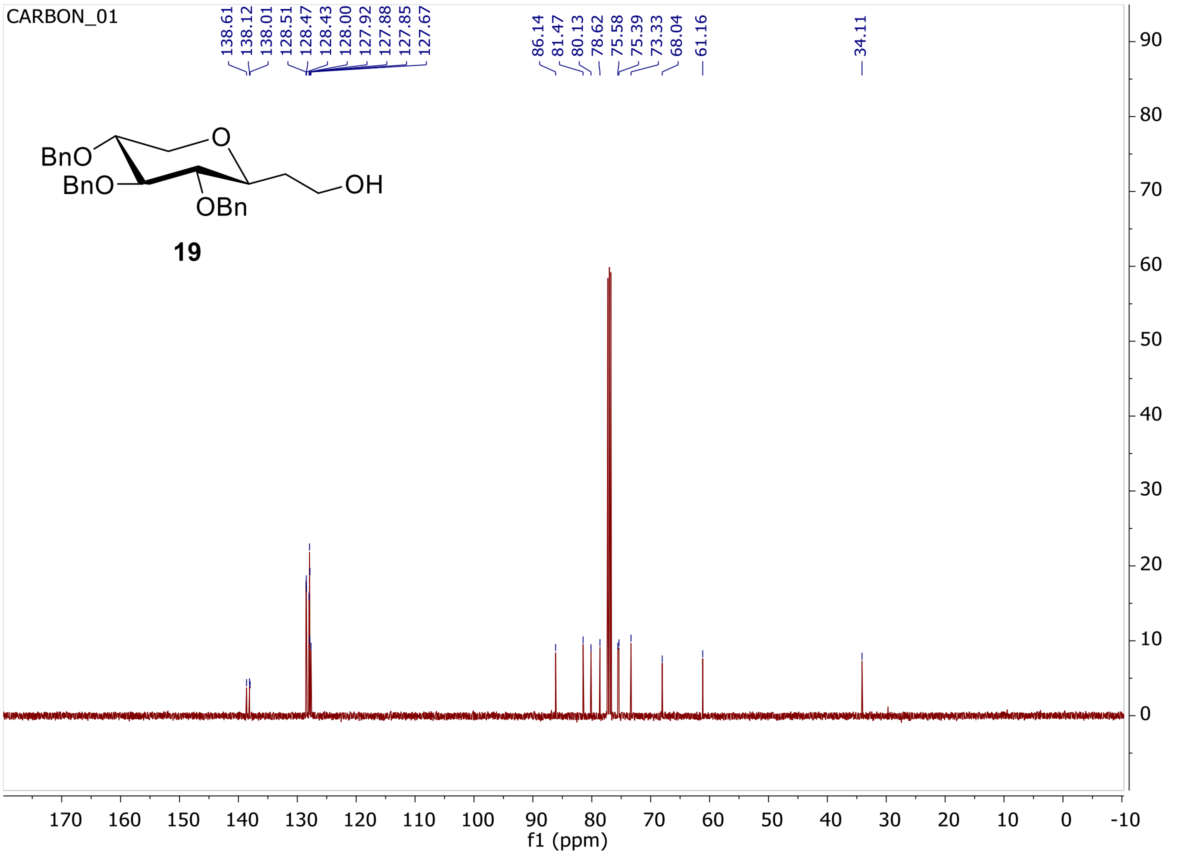

Figure S14. ^{13}C NMR spectrum of **19**

Table S1: ^{1}H-NMR analysis of crude product from batch and flow reactions of xylopyranose derivative **8** and glucopyranose derivative **3**

Entry	Reactant	Conditions	Wittig Reaction Products (ratio)	C-Glycosyl Products (ratio)
1	**8**	Batch: Ph_3PCHCO_2Et (3 eq), toluene, 90 °C, 18 h	**9-(E):9-(Z)** (1:0.3)	Not observed
2	**8**	Flow: Ph_3PCHCO_2Et (5 eq), toluene, 180 °C (reactor 1), 130 °C (reactor 2), 90 mins	**9-(E):9-(Z)** Not observed	**10:12** (1:0.14)
3	**3**	Batch: Ph_3PCHCO_2Et (3 eq), toluene, 110 °C, 8 h	**3** present & trace of **11-(E)**	N/A
4	**3**	Flow: Ph_3PCHCO_2Et (5 eq), toluene, 200 °C (reactor 1), 145 °C (reactor 2), 80 mins	**11-(E) : 7b** (0.25: 1.0)	
